# Supplementary material for: Network community structure of substorms using SuperMAG magnetometers
Source: Nat Commun. 2021 Mar 23;12:1842. doi: 10.1038/s41467-021-22112-4 (PMC7988152; doi:10.1038/s41467-021-22112-4)
Supplement: Supplementary file 1 — Supplementary Information [file 41467_2021_22112_MOESM1_ESM.pdf]

Supplementary Information: Network Community Structure of Substorms using SuperMAG Magnetometers

L.Orr[1,*], S.C.Chapman[1], J.W.Gjerloev[2,3], and W. Guo[4,5]

[1]Centre for Fusion, Space and Astrophysics, University of Warwick, UK
[2]Applied Physics Laboratory- John Hopkins University, Laurel, Maryland, USA
[3]Birkeland Centre, University of Bergen, Norway
[4]School of Aerospace, Cranfield University, UK
[5]Alan Turing Institute, UK
[*]l.orr1@lancaster.ac.uk

Contents of Supplementary Information

1. Supplementary Note 1, Supplementary Figures 1 to 13
2. Supplementary Note 2, Supplementary Figures 14 to 24
3. Supplementary Note 3, Supplementary Figure 25
4. Supplementary Note 4, Supplementary Figures 26 to 27
5. Supplementary Note 5, Supplementary Figure 28
6. Supplementary Note 6, Supplementary Figures 29-32
7. Supplementary Note 7, Glossary with Supplementary Figures 33-35
8. Supplementary Note 8, List of Substorms used in study, Supplementary Tables 1 to 3

Introduction

In Supplementary Note 1 we present examples of four substorm events plotted in the same format as the main text and auxiliary plots for all these events including that presented in the main text (Supplementary Figures 1-13). In Supplementary Note 2 we vary the community detection methods and the parameters used to construct the raw network (Supplementary Figures 14-24). In Supplementary Note 3 we compare the substorm modularity from these events to that of random networks (Supplementary Figure 25). Supplementary Note 4, Supplementary Figures 26- 27 plot the magnetometer spatial coverage and separation distances of each of the 41 events at the time of substorm onset. Supplementary Note 5, Supplementary Figure 28 is an example of canonical cross correlation on model data. Supplementary Note 6, Supplementary Figures 29-32 show figures of the community structure for two flow burst events. Supplementary Note 7, Supplementary Figures 33-36 accompany a glossary. Supplementary Note 8 contains Supplementary tables 1-3 with the substorm timings used for this study.

Supplementary Note 1

Supplementary Figures 1-13 show individual examples of the community structure for different events, using the edge betweenness algorithm[1], as used within the main text. Supplementary Figure 1 presents the 16/03/1997 substorm (the example used in the main text) in the same format as Figure 2, main text except that it overlays snapshots of the network on the superMAG polar plot. We present the sequence of plot as shown in Figure 1, 2 (main text) and Supplementary Figure 1 for four additional events: a substorm on the 07/01/1997 (2-4), 06/09/1997 (5-7) and 06/01/1998 (8-10) and 20/01/1998 (11-13).

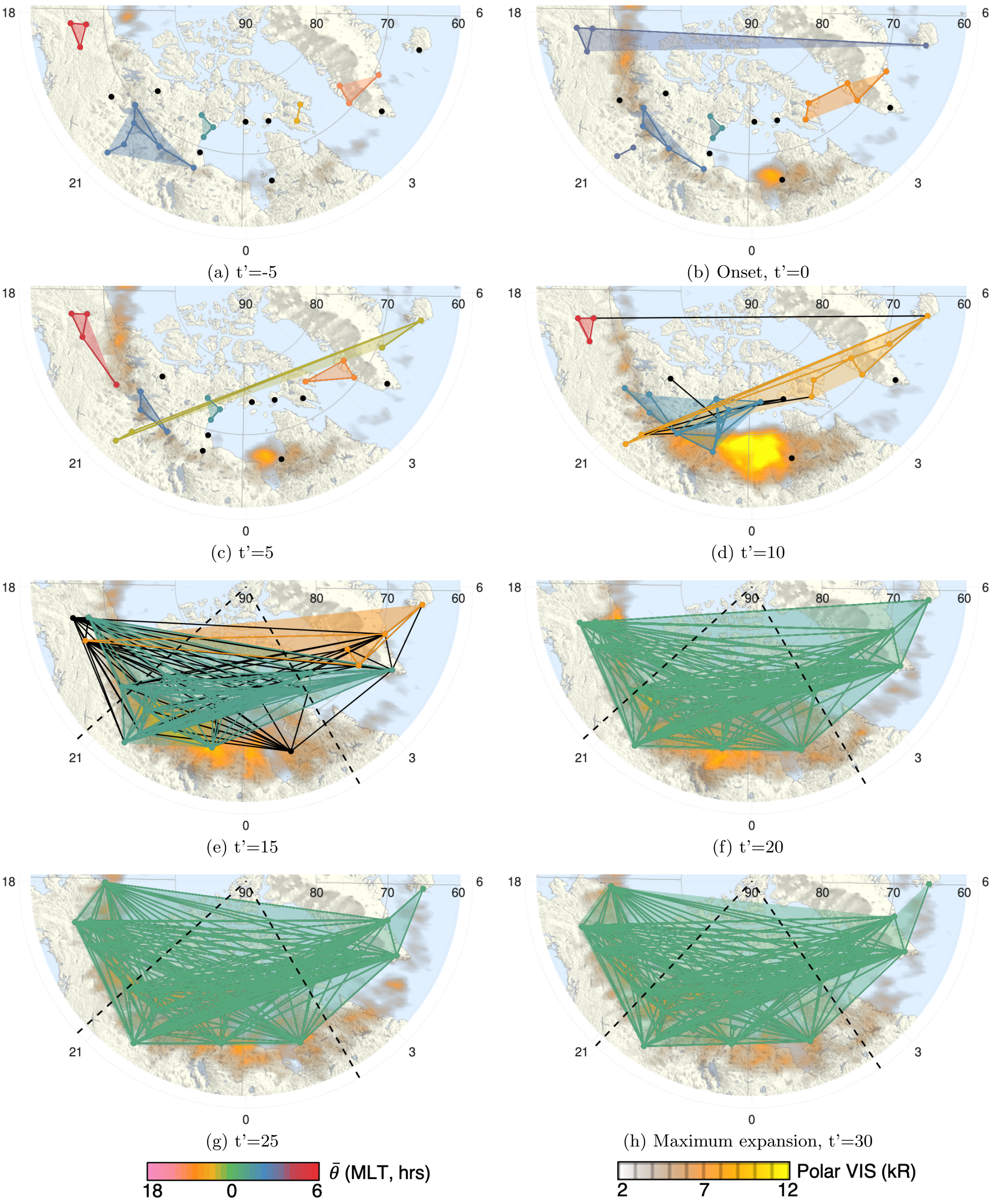

Supplementary Figure 1. Community structure snapshots of an example substorm network in normalized time, t'. The community structure of a substorm on the 16/03/1997 (the same example as used in the main text), which is plotted in the same format as Figure 2, main text, except that all network connections are plotted (and magnetometer vectors are not plotted). Polar plots are in magnetic coordinates centered at the magnetic pole, where magnetic local time (MLT, hrs) increases clockwise, with midnight located at the bottom (MLT=0 hrs). Maps show the nightside from dusk (MLT=18 hrs) to dawn (MLT=6 hrs) and 60-90° magnetic latitude. The colorbars at the bottom of the figure represent the MLT of the centroid ($\bar{\theta}(t')$) of each community, and polar VIS data from left to right respectively. The network connections are color coded using the left and match those of panels c-d in Figure 1, main text. Each subplot (a-h) represents a snapshot of the community structure in intervals of 5 normalized minutes from before onset (panel a, $t' = -5$) to the time of maximum expansion (panel h, $t' = 30$), corresponding to the times in Figure 1, main text. The circles represent ground magnetometers with the lines representing network connections. Black magnetometers/connections are not part of a community. The black dashed lines, in panels e-h, are the locations of the auroral bulge found from auroral images. The networks are overplotted on maps provided by superMAG[2] containing polar VIS data[3] in kR, matching the right colorbar. There is a clear change in community structure from $t' \sim 10$, with many more inter-community connections forming at $t' \sim 15$, and merging into a single community at $t' \sim 20$.

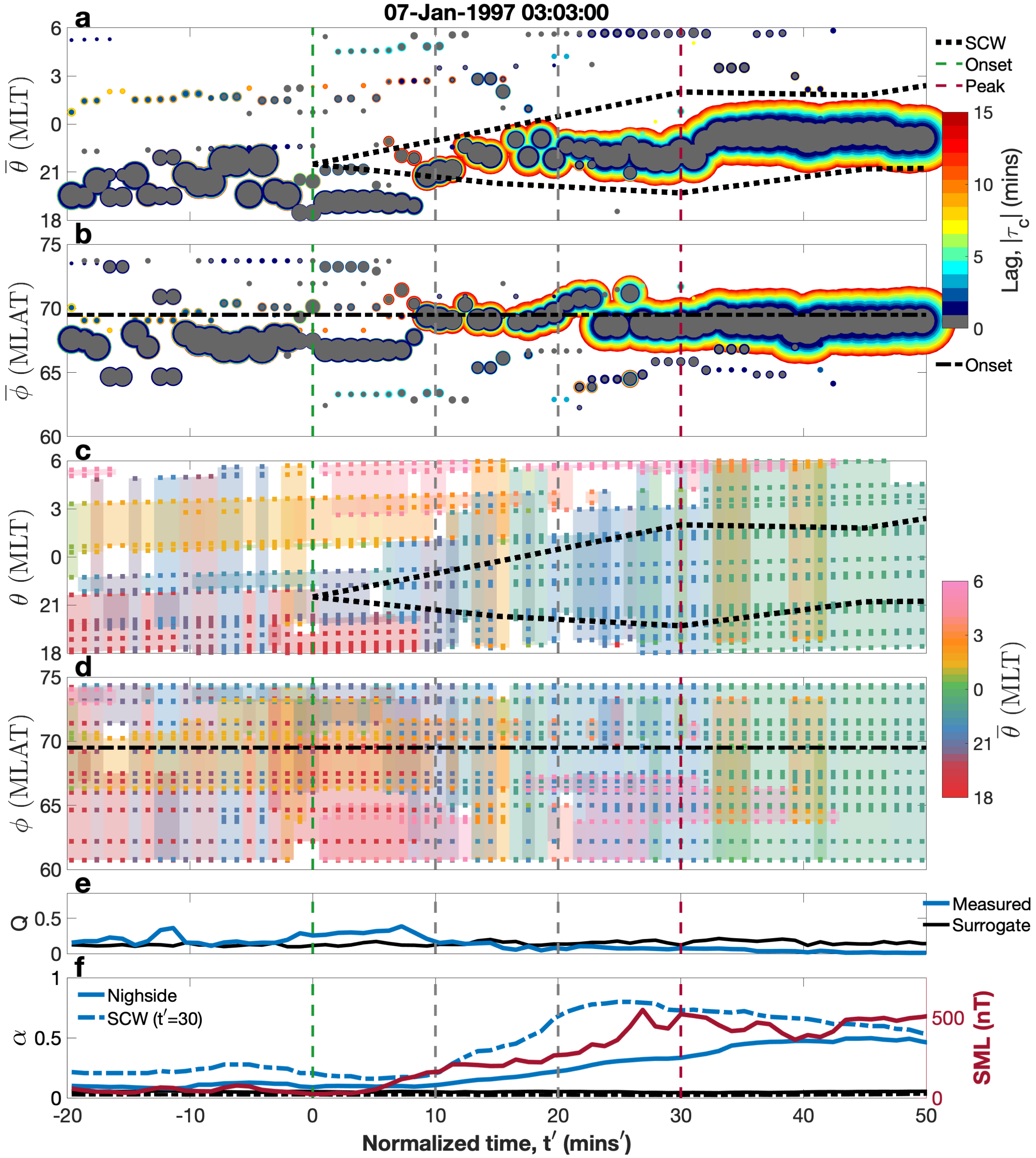

Supplementary Figure 2. Community structure of an example substorm. The community structure of a substorm on 07/01/1997, plotted in the same format as Figure 1, main text (which shows a different event, 16/03/1997). The abscissa of all panels is normalized time ($t' = 0$ is onset (dashed green line) and $t' = 30$ (dashed purple line) is the time of maximum auroral bulge expansion). Vertical grey dashed lines show 10 normalized minute intervals within the expansion phase. Panels a-b plots individual communities as circles where the size of the circle reflects the number of connections within the community. The ordinate plots the mean magnetic local time/ latitude (MLT/MLAT in hrs/degrees) of the community, $\bar{\theta}_x(t')$ and $\bar{\phi}_x(t')$, and the color indicates the proportion of connections with each time lag, $|\tau_c|$. The dashed lines overplotted are the edges of the auroral bulge (MLT) and the onset location (MLAT), found from auroral images. Panels c-d show the spatial extent of each community, where the dots are the magnetometer locations and the shading is the extent. Color represents the mean MLT of the stations contained within each community, $\bar{\theta}_x(t')$. Panel e plots the modularity, Q, (blue line) and the random phase surrogate (black line). Panel f plots the normalized number of connections, $\alpha(t')$, both within the nightside (solid blue) and within the SCW (dashed blue), as well as their surrogates (solid and dashed black respectively, both near zero throughout). The right ordinate plots (negative) SML (red). Following onset, Panels a and b show that there is a dominant community but additionally several smaller communities which persist throughout the substorm. The modularity plotted in panel e drops from ~ 0.5 before the event to almost zero by $t' \sim 20$. This plot used the edge betweenness community detection algorithm as in the main text.

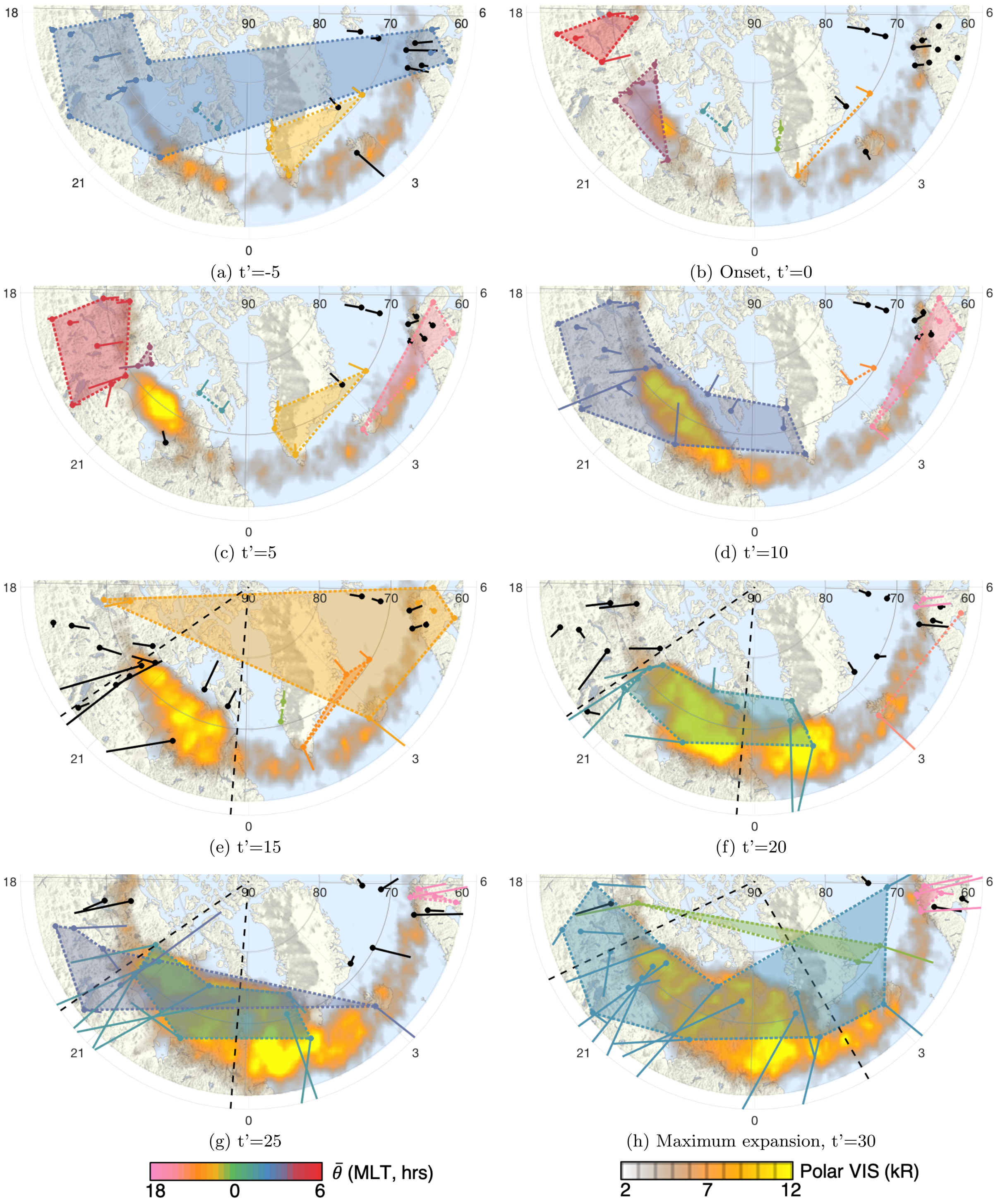

Supplementary Figure 3. Community structure snapshots of an example substorm vectors. The community structure of a substorm on 07/01/1997, plotted in the same format as Figure 2, main text (which shows a different event, 16/03/1997). Polar plots are in magnetic coordinates centered at the magnetic pole, where magnetic local time (MLT, hrs) increases clockwise, with midnight located at the bottom (MLT=0 hrs). Maps show the nightside from dusk (MLT=18 hrs) to dawn (MLT=6 hrs) and 60-90° magnetic latitude. Plotted are the magnetic field perturbation vectors (North and East components, $B_{N,E}$, measured in nT) for a substorm on 16/03/1997. The colorbars at the bottom of the figure represent the MLT of the centroid ($\bar{\theta}(t')$) of each community, and polar VIS data from left to right respectively. The vectors are color coded using the left and match those of panels c-d in Supplementary Figure 2. Each subplot (a-h) represents a snapshot of the community structure in intervals of 5 normalized minutes from before onset (panel a, $t' = -5$) to the time of maximum expansion (panel h, $t' = 30$), corresponding to the times in Supplementary Figure 2. The circles represent ground magnetometers with the line representing the $B_{N,E}$ vector. Black magnetometers are not part of a community. The black dashed lines, in panels e-h, are the locations of the auroral bulge found from auroral images. The vectors are overplotted on maps provided by superMAG[2] containing polar VIS data[3] in kR, matching the right colorbar. Several smaller communities are maintained throughout but the network tends towards one main community. This plot used the edge betweenness community detection algorithm as in the main text.

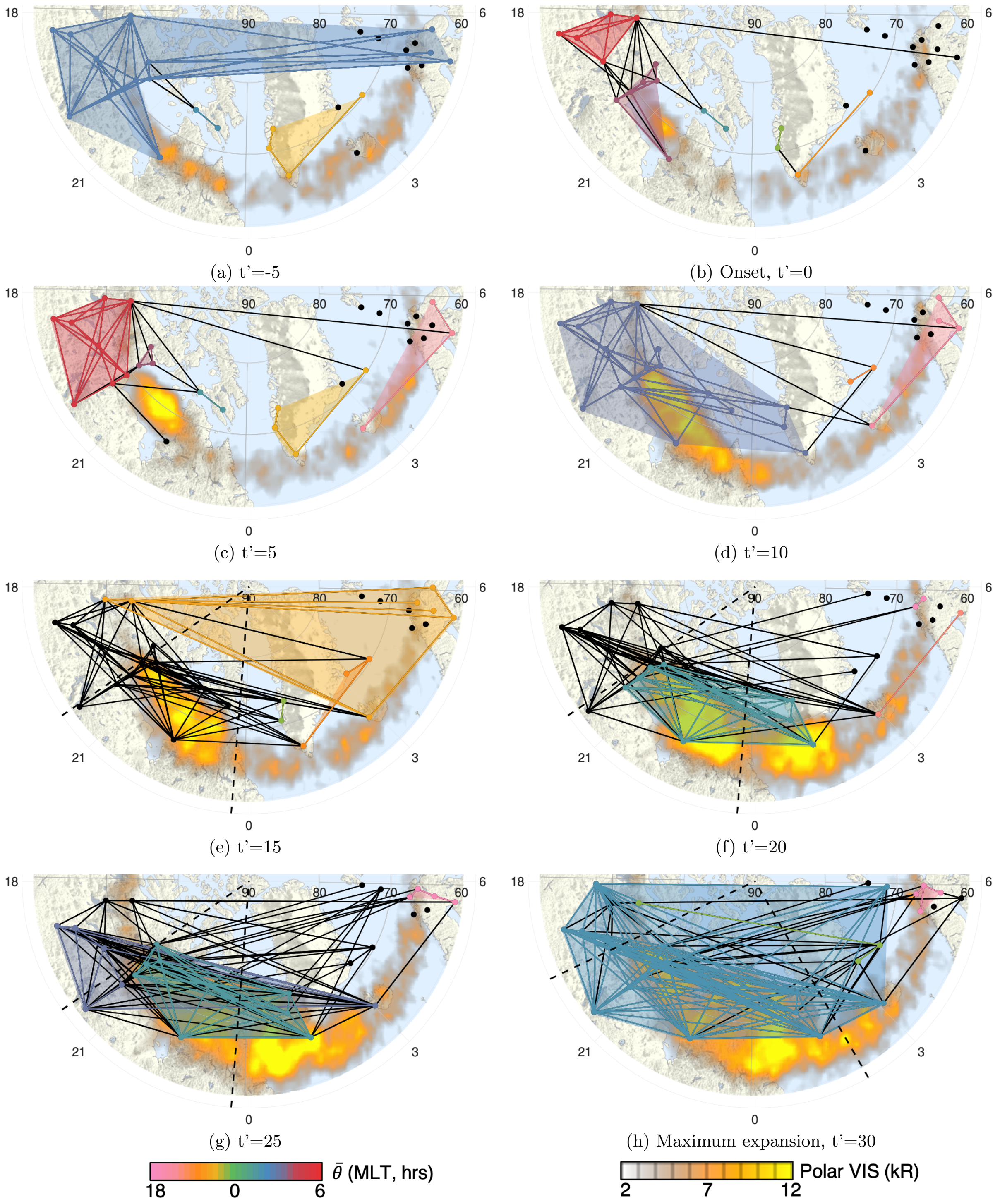

Supplementary Figure 4. Community structure snapshots of an example substorm network in normalized time, t'. The community structure of a substorm on 07/01/1997, plotted in the same format as Supplementary Figure 1 (which shows a different event, 16/03/1997). Polar plots are in magnetic coordinates centered at the magnetic pole, where magnetic local time (MLT, hrs) increases clockwise, with midnight located at the bottom (MLT=0 hrs). Maps show the nightside from dusk (MLT=18 hrs) to dawn (MLT=6 hrs) and 60-90° magnetic latitude. The colorbars at the bottom of the figure represent the MLT of the centroid ($\bar{\theta}(t')$) of each community, and polar VIS data from left to right respectively. The network connections are color coded using the left and match those of panels c-d in Supplementary Figure 2. Each subplot (a-h) represents a snapshot of the community structure in intervals of 5 normalized minutes from before onset (panel a, $t' = -5$) to the time of maximum expansion (panel h, $t' = 30$), corresponding to the times in Supplementary Figure 2. The circles represent ground magnetometers with the lines representing network connections. Black magnetometers/connections are not part of a community. The black dashed lines, in panels e-h, are the locations of the auroral bulge found from auroral images. The networks are overplotted on maps provided by superMAG[2] containing polar VIS data[3] in kR, matching the right colorbar. The smaller communities transition into a dominant community, with several smaller communities persisting throughout but they are connected via many inter-community edges. This plot used the edge betweenness community detection algorithm as in the main text.

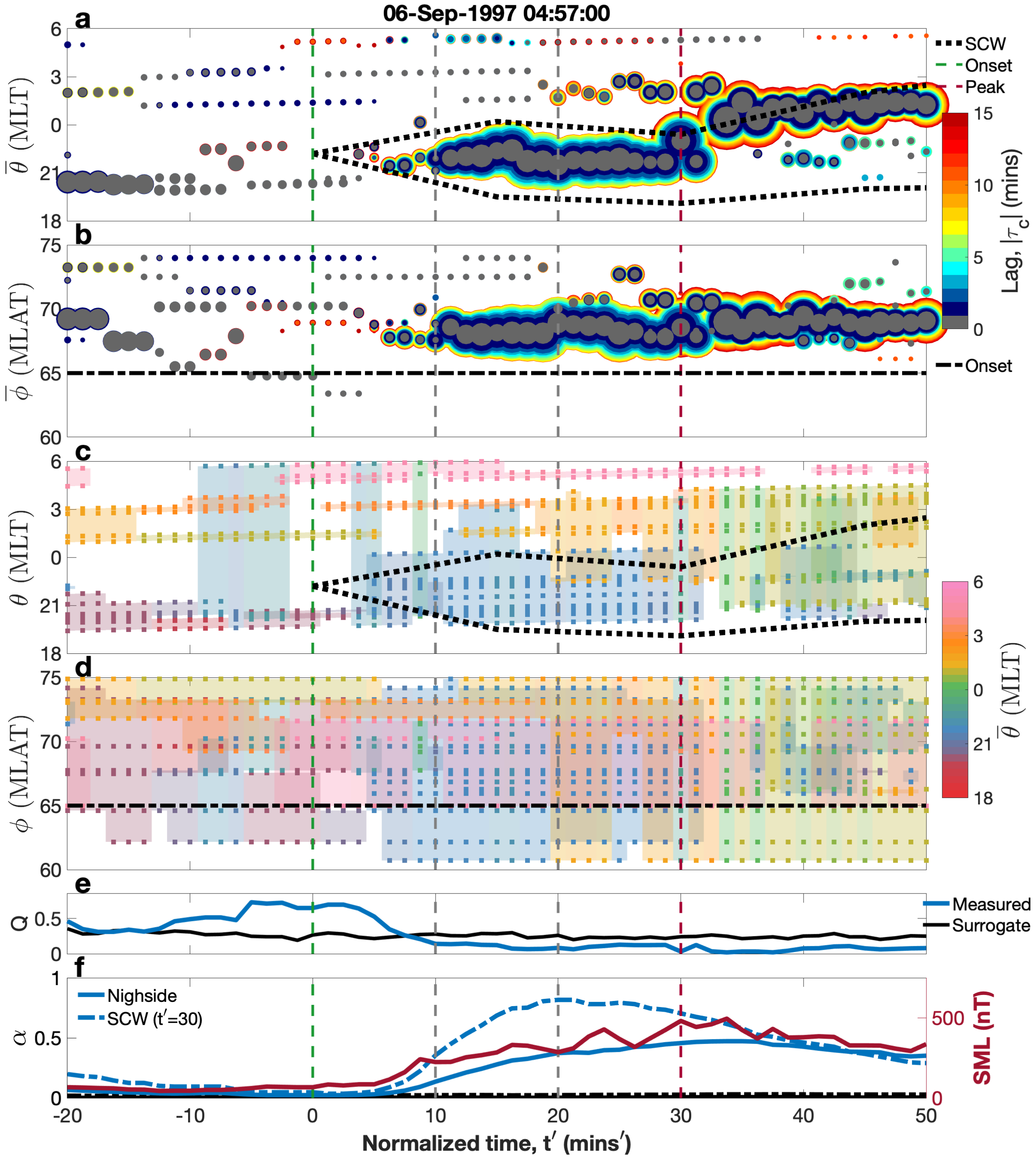

Supplementary Figure 5. Community structure of an example substorm. The community structure of a substorm on 06/09/1997, plotted in the same format as Figure 1, main text (which shows a different event, 16/03/1997). The abscissa of all panels is normalized time ($t' = 0$ is onset (dashed green line) and $t' = 30$ (dashed purple line) is the time of maximum auroral bulge expansion). Vertical grey dashed lines show 10 normalized minute intervals within the expansion phase. Panels a-b plots individual communities as circles where the size of the circle reflects the number of connections within the community. The ordinate plots the mean magnetic local time/ latitude (MLT/MLAT in hrs/degrees) of the community, $\bar{\theta}_x(t')$ and $\bar{\phi}_x(t')$, and the color indicates the proportion of connections with each time lag, $|\tau_c|$. The dashed lines overplotted are the edges of the auroral bulge (MLT) and the onset location (MLAT), found from auroral images. Panels c-d show the spatial extent of each community, where the dots are the magnetometer locations and the shading is the extent. Color represents the mean MLT of the stations contained within each community, $\bar{\theta}_x(t')$. Panel e plots the modularity, Q, (blue line) and the random phase surrogate (black line). Panel f plots the normalized number of connections, $\alpha(t')$, both within the nightside (solid blue) and within the SCW (dashed blue), as well as their surrogates (solid and dashed black respectively, both near zero throughout). The right ordinate plots (negative) SML (red). Following onset, Panels a and b show that there is a dominant community but, additionally, several smaller communities which persist throughout the substorm. The modularity plotted in panel e drops from ~ 0.7 before the event to almost zero by $t' \sim 10$. This plot used the edge betweenness community detection algorithm as in the main text.

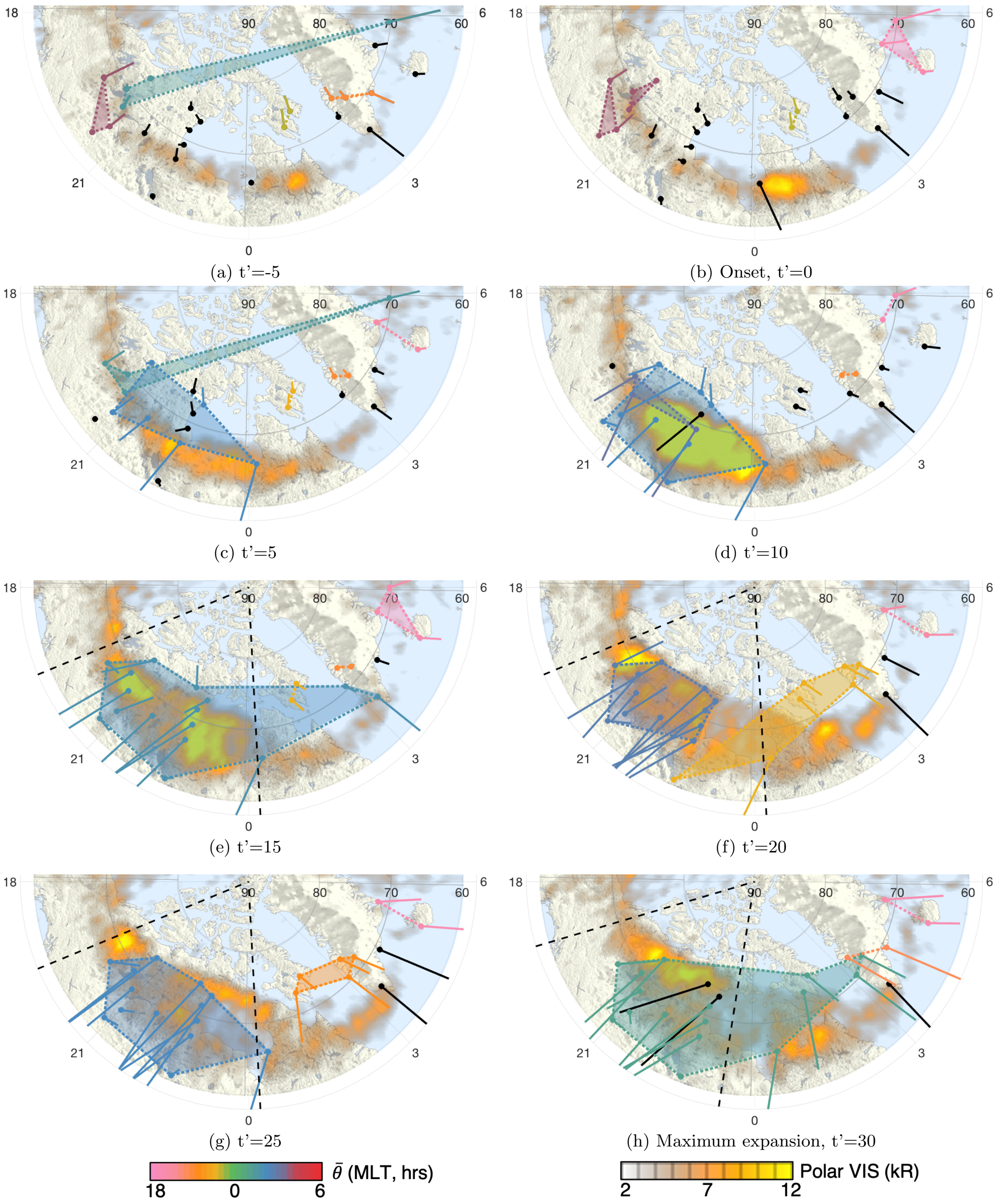

(a) t'=-5
(b) Onset, t'=0
(c) t'=5
(d) t'=10
(e) t'=15
(f) t'=20
(g) t'=25
(h) Maximum expansion, t'=30

Supplementary Figure 6. Community structure snapshots of an example substorm vectors in normalized time t'. The community structure of a substorm on 06/09/1997, plotted in the same format as Figure 2, main text (which shows a different event, 16/03/1997). Polar plots are in magnetic coordinates centered at the magnetic pole, where magnetic local time (MLT, hrs) increases clockwise, with midnight located at the bottom (MLT=0 hrs). Maps show the nightside from dusk (MLT=18 hrs) to dawn (MLT=6 hrs) and 60-90° magnetic latitude. Plotted are the magnetic field perturbation vectors (North and East components, $B_{N,E}$, measured in nT) for a substorm on 16/03/1997. The colorbars at the bottom of the figure represent the MLT of the centroid ($\bar{\theta}(t')$) of each community, and polar VIS data from left to right respectively. The vectors are color coded using the left and match those of panels c-d in Supplementary Figure 5. Each subplot (a-h) represents a snapshot of the community structure in intervals of 5 normalized minutes from before onset (panel a, $t' = -5$) to the time of maximum expansion (panel h, $t' = 30$), corresponding to the times in Supplementary Figure 5. The circles represent ground magnetometers with the line representing the $B_{N,E}$ vector. Black magnetometers are not part of a community. The black dashed lines, in panels e-h, are the locations of the auroral bulge found from auroral images. The vectors are overplotted on maps provided by superMAG[2] containing polar VIS data[3] in kR, matching the right colorbar. Several smaller communities are maintained throughout the substorm but the network tends towards one main community. This plot used the edge betweenness community detection algorithm as in the main text.

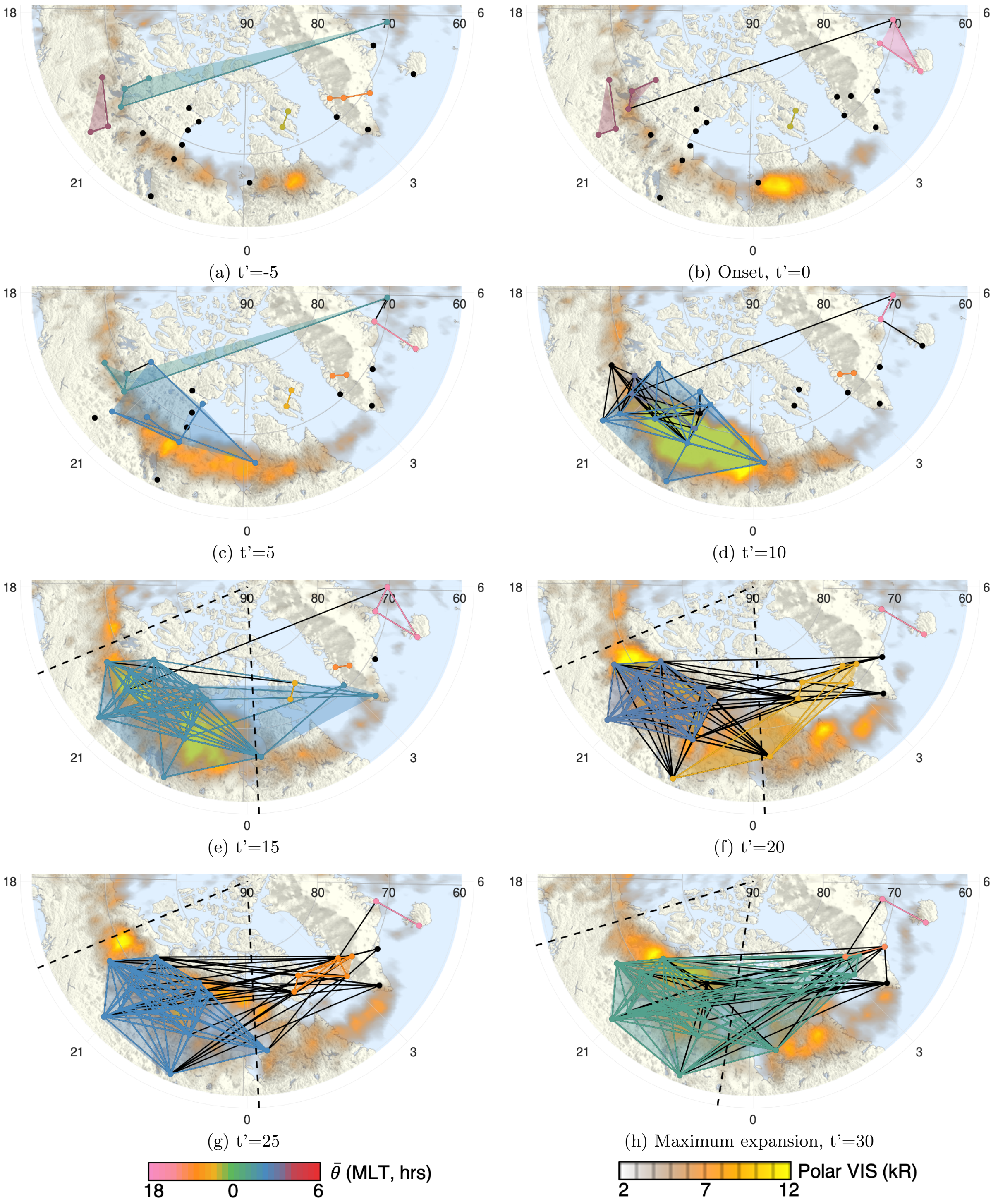

(a) t'=-5
(b) Onset, t'=0
(c) t'=5
(d) t'=10
(e) t'=15
(f) t'=20
(g) t'=25
(h) Maximum expansion, t'=30

Supplementary Figure 7. Community structure snapshots of an example substorm network in normalized time t'. The community structure of a substorm on 06/09/1997, plotted in the same format as Supplementary Figure 1 (which shows a different event, 16/03/1997). Polar plots are in magnetic coordinates centered at the magnetic pole, where magnetic local time (MLT, hrs) increases clockwise, with midnight located at the bottom (MLT=0 hrs). Maps show the nightside from dusk (MLT=18 hrs) to dawn (MLT=6 hrs) and 60-90° magnetic latitude. The colorbars at the bottom of the figure represent the MLT of the centroid ($\bar{\theta}(t')$) of each community, and polar VIS data from left to right respectively. The network connections are color coded using the left and match those of panels c-d in Supplementary Figure 5. Each subplot (a-h) represents a snapshot of the community structure in intervals of 5 normalized minutes from before onset (panel a, $t' = -5$) to the time of maximum expansion (panel h, $t' = 30$), corresponding to the times in Supplementary Figure 5. The circles represent ground magnetometers with the lines representing network connections. Black magnetometers/connections are not part of a community. The black dashed lines, in panels e-h, are the locations of the auroral bulge found from auroral images. The networks are overplotted on maps provided by superMAG[2] containing polar VIS data[3] in kR, matching the right colorbar. The smaller communities transition into a dominant community, with several smaller communities persisting throughout the substorm but they are connected via many inter-community edges. This plot used the edge betweenness community detection algorithm as in the main text.

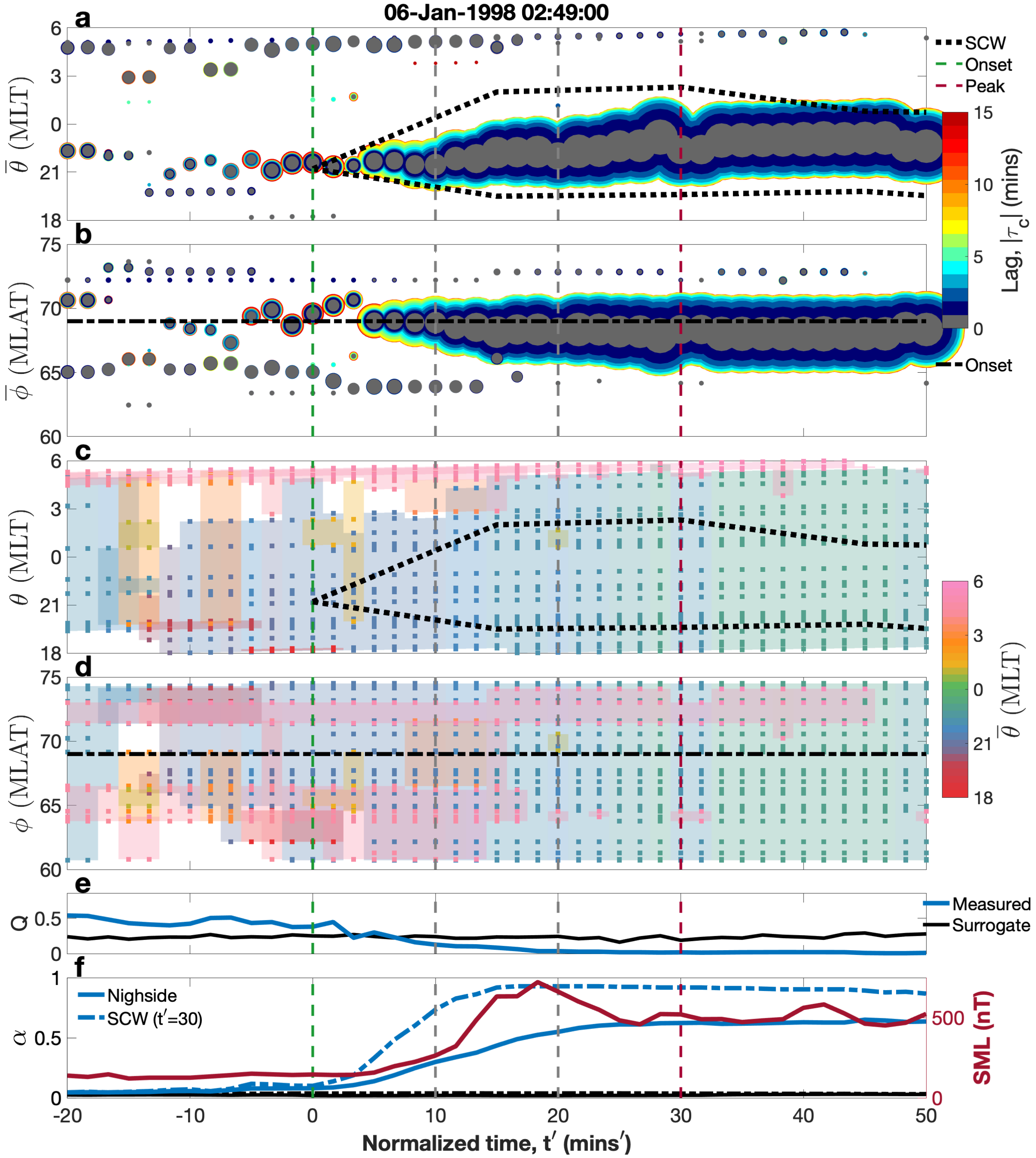

Supplementary Figure 8. Community structure of an example substorm. The community structure of a substorm on 06/01/1998, plotted in the same format as Figure 1, main text (which shows a different event, 16/03/1997). The abscissa of all panels is normalized time ($t' = 0$ is onset (dashed green line) and $t' = 30$ (dashed purple line) is the time of maximum auroral bulge expansion). Vertical grey dashed lines show 10 normalized minute intervals within the expansion phase. Panels a-b plots individual communities as circles where the size of the circle reflects the number of connections within the community. The ordinate plots the mean magnetic local time/ latitude (MLT/MLAT in hrs/degrees) of the community, $\bar{\theta}_x(t')$ and $\bar{\phi}_x(t')$, and the color indicates the proportion of connections with each time lag, $|\tau_c|$. The dashed lines overplotted are the edges of the auroral bulge (MLT) and the onset location (MLAT), found from auroral images. Panels c-d show the spatial extent of each community, where the dots are the magnetometer locations and the shading is the extent. Color represents the mean MLT of the stations contained within each community, $\bar{\theta}_x(t')$. Panel e plots the modularity, Q, (blue line) and the random phase surrogate (black line). Panel f plots the normalized number of connections, $\alpha(t')$, both within the nightside (solid blue) and within the SCW (dashed blue), as well as their surrogates (solid and dashed black respectively, both near zero throughout). The right ordinate plots (negative) SML (red). Following onset, Panels a and b show that there is a dominant community but also a single smaller community (at ~ 6 MLT) which persist throughout the substorm. The modularity plotted in panel e drops from ~ 0.5 before the event to almost zero by $t' \sim 20$. This plot used the edge betweenness community detection algorithm as in the main text.

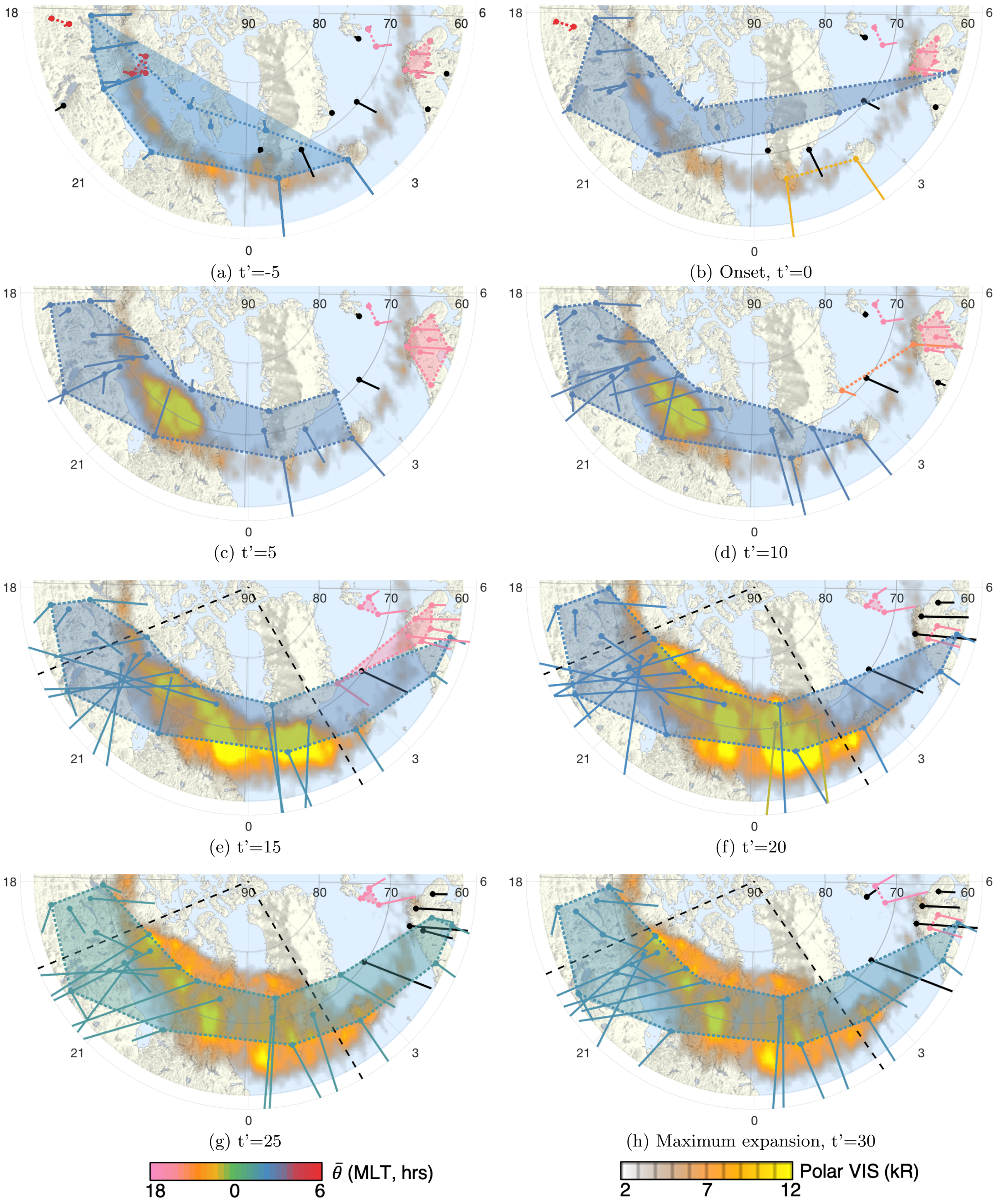

Supplementary Figure 9. Community structure snapshots of an example substorm vectors in normalized time t'. The community structure of a substorm on 06/01/1998, plotted in the same format as in Figure 2, main text (which shows a different event, 16/03/1997). Polar plots are in magnetic coordinates centered at the magnetic pole, where magnetic local time (MLT, hrs) increases clockwise, with midnight located at the bottom (MLT=0 hrs). Maps show the nightside from dusk (MLT=18 hrs) to dawn (MLT=6 hrs) and 60-90° magnetic latitude. Plotted are the magnetic field perturbation vectors (North and East components, $B_{N,E}$, measured in nT) for a substorm on 16/03/1997. The colorbars at the bottom of the figure represent the MLT of the centroid ($\bar{\theta}(t')$) of each community, and polar VIS data from left to right respectively. The vectors are color coded using the left and match those of panels c-d in Supplementary Figure 8. Each subplot (a-h) represents a snapshot of the community structure in intervals of 5 normalized minutes from before onset (panel a, $t' = -5$) to the time of maximum expansion (panel h, $t' = 30$), corresponding to the times in Supplementary Figure 8. The circles represent ground magnetometers with the line representing the $B_{N,E}$ vector. Black magnetometers are not part of a community. The black dashed lines, in panels e-h, are the locations of the auroral bulge found from auroral images. The vectors are overplotted on maps provided by superMAG[2] containing polar VIS data[3] in kR, matching the right colorbar. A few smaller communities are maintained throughout expansion (at ~ 6 MLT) but the network tends towards one main community. This plot used the edge betweenness community detection algorithm as in the main text.

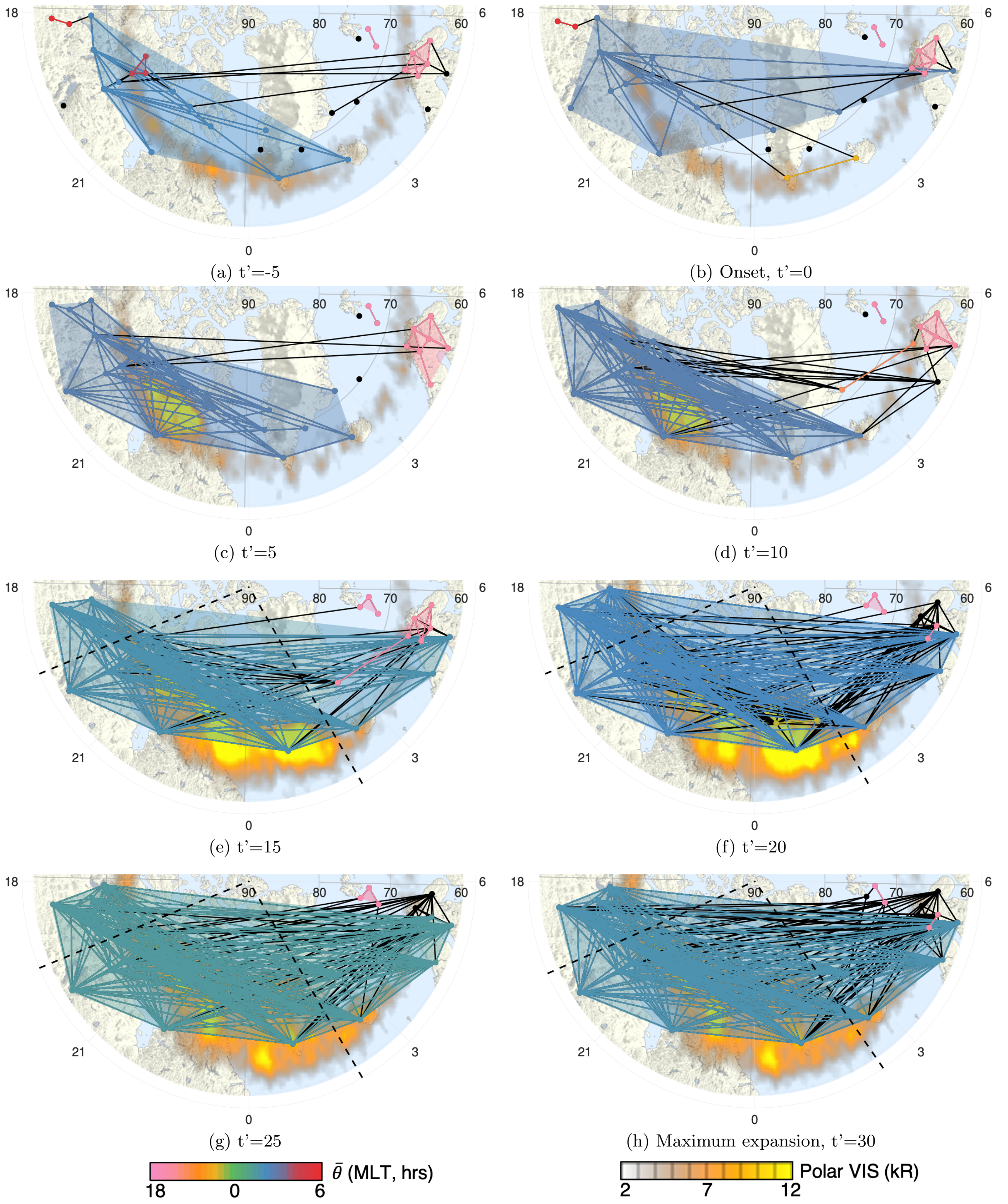

Supplementary Figure 10. Community structure snapshots of an example substorm network in normalized time t'. The community structure of a substorm on 06/01/1998, plotted in the same format as Supplementary Figure 1 (which shows a different event, 16/03/1997). Polar plots are in magnetic coordinates centered at the magnetic pole, where magnetic local time (MLT, hrs) increases clockwise, with midnight located at the bottom (MLT=0 hrs). Maps show the nightside from dusk (MLT=18 hrs) to dawn (MLT=6 hrs) and 60-90° magnetic latitude. The colorbars at the bottom of the figure represent the MLT of the centroid ($\bar{\theta}(t')$) of each community, and polar VIS data from left to right respectively. The network connections are color coded using the left and match those of panels c-d in Supplementary Figure 8. Each subplot (a-h) represents a snapshot of the community structure in intervals of 5 normalized minutes from before onset (panel a, $t' = -5$) to the time of maximum expansion (panel h, $t' = 30$), corresponding to the times in Supplementary Figure 8. The circles represent ground magnetometers with the lines representing network connections. Black magnetometers/connections are not part of a community. The black dashed lines, in panels e-h, are the locations of the auroral bulge found from auroral images. The networks are overplotted on maps provided by superMAG[2] containing polar VIS data[3] in kR, matching the right colorbar. The main community expands eastward to form a large global community, with a $1-2$ smaller communities around dawn but between the communities there are many connections. This plot used the edge betweenness community detection algorithm as in the main text.

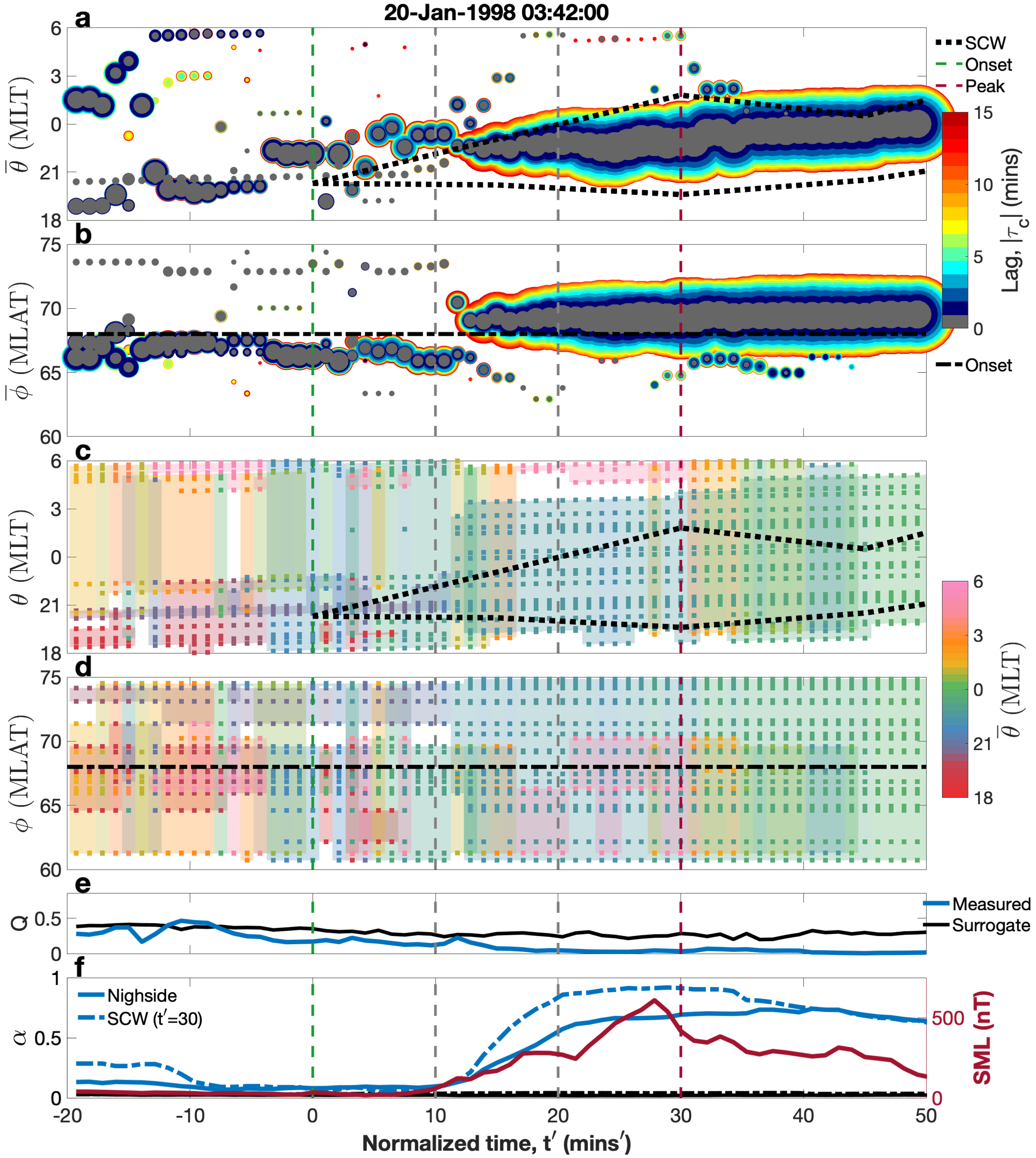

Supplementary Figure 11. Community structure of an example substorm. The community structure of a substorm on 20/01/1998, plotted in the same format as Figure 1, main text (which shows a different event, 16/03/1997). The abscissa of all panels is normalized time ($t' = 0$ is onset (dashed green line) and $t' = 30$ (dashed purple line) is the time of maximum auroral bulge expansion). Vertical grey dashed lines show 10 normalized minute intervals within the expansion phase. Panels a-b plots individual communities as circles where the size of the circle reflects the number of connections within the community. The ordinate plots the mean magnetic local time/ latitude (MLT/MLAT in hrs/degrees) of the community, $\bar{\theta}_x(t')$ and $\bar{\phi}_x(t')$, and the color indicates the proportion of connections with each time lag, $|\tau_c|$. The dashed lines overplotted are the edges of the auroral bulge (MLT) and the onset location (MLAT), found from auroral images. Panels c-d show the spatial extent of each community, where the dots are the magnetometer locations and the shading is the extent. Color represents the mean MLT of the stations contained within each community, $\bar{\theta}_x(t')$. Panel e plots the modularity, Q, (blue line) and the random phase surrogate (black line). Panel f plots the normalized number of connections, $\alpha(t')$, both within the nightside (solid blue) and within the SCW (dashed blue), as well as their surrogates (solid and dashed black respectively, both near zero throughout). The right ordinate plots (negative) SML (red). Following onset, Panels a and b show that there is a dominant community but also a single smaller community which persists throughout expansion. The modularity plotted in panel e drops from ~ 0.5 before the event to almost zero by $t' \sim 20$. This plot used the edge betweenness community detection algorithm as in the main text.

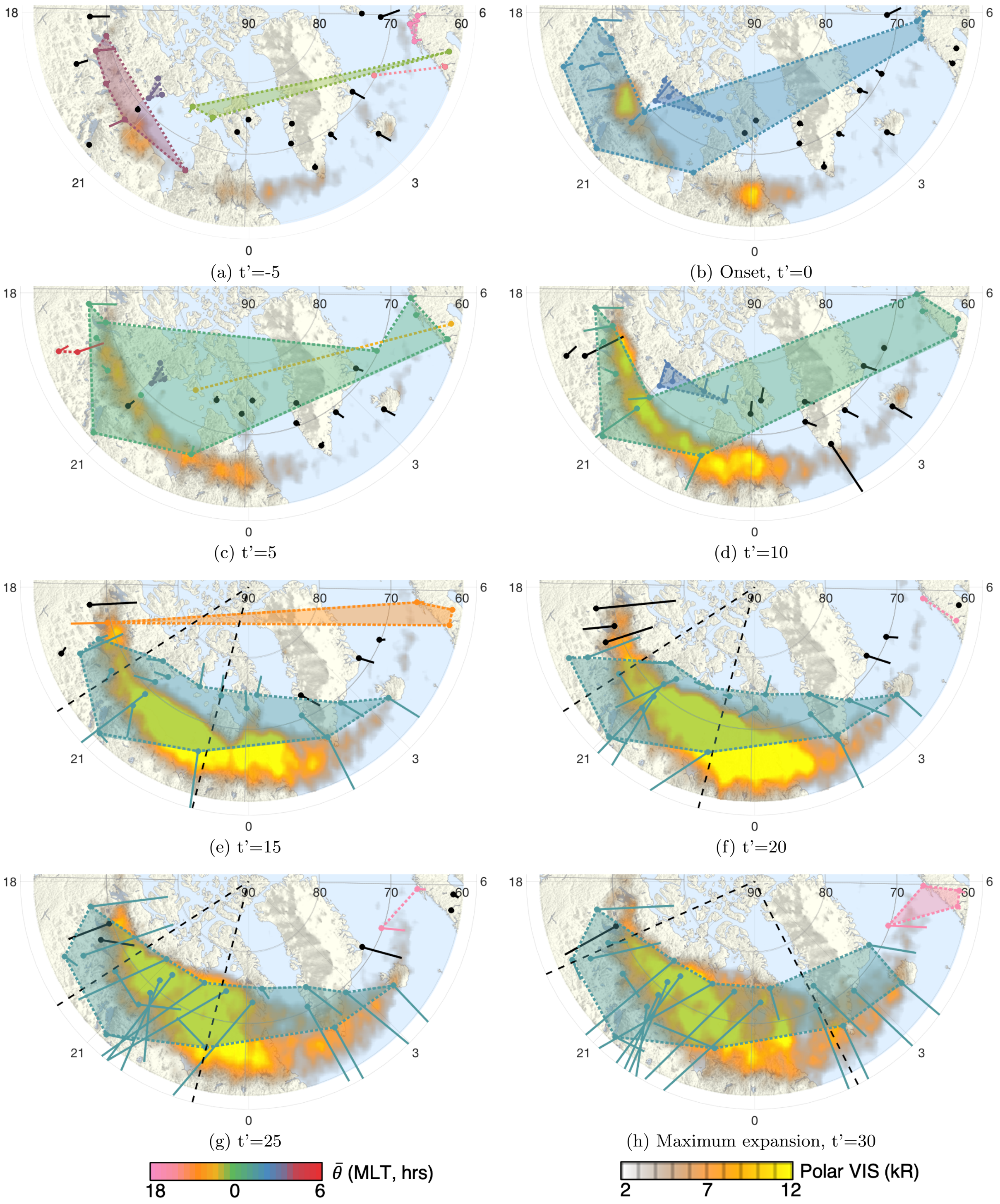

Supplementary Figure 12. Community structure snapshots of an example substorm vectors in normalized time t'. The community structure of a substorm on 20/01/1998, plotted in the same format as Figure 2, main text (which shows a different event, 16/03/1997). Polar plots are in magnetic coordinates centered at the magnetic pole, where magnetic local time (MLT, hrs) increases clockwise, with midnight located at the bottom (MLT=0 hrs). Maps show the nightside from dusk (MLT=18 hrs) to dawn (MLT=6 hrs) and 60-90° magnetic latitude. Plotted are the magnetic field perturbation vectors (North and East components, $B_{N,E}$, measured in nT) for a substorm on 16/03/1997. The colorbars at the bottom of the figure represent the MLT of the centroid ($\bar{\theta}(t')$) of each community, and polar VIS data from left to right respectively. The vectors are color coded using the left and match those of panels c-d in Supplementary Figure 11. Each subplot (a-h) represents a snapshot of the community structure in intervals of 5 normalized minutes from before onset (panel a, $t' = -5$) to the time of maximum expansion (panel h, $t' = 30$), corresponding to the times in Supplementary Figure 11. The circles represent ground magnetometers with the line representing the $B_{N,E}$ vector. Black magnetometers are not part of a community. The black dashed lines, in panels e-h, are the locations of the auroral bulge found from auroral images. The vectors are overplotted on maps provided by superMAG[2] containing polar VIS data[3] in kR, matching the right colorbar. The smaller communities transition into a dominant community but there remains a single smaller community (at ~ 6 MLT) which persists throughout. This plot used the edge betweenness community detection algorithm as in the main text.

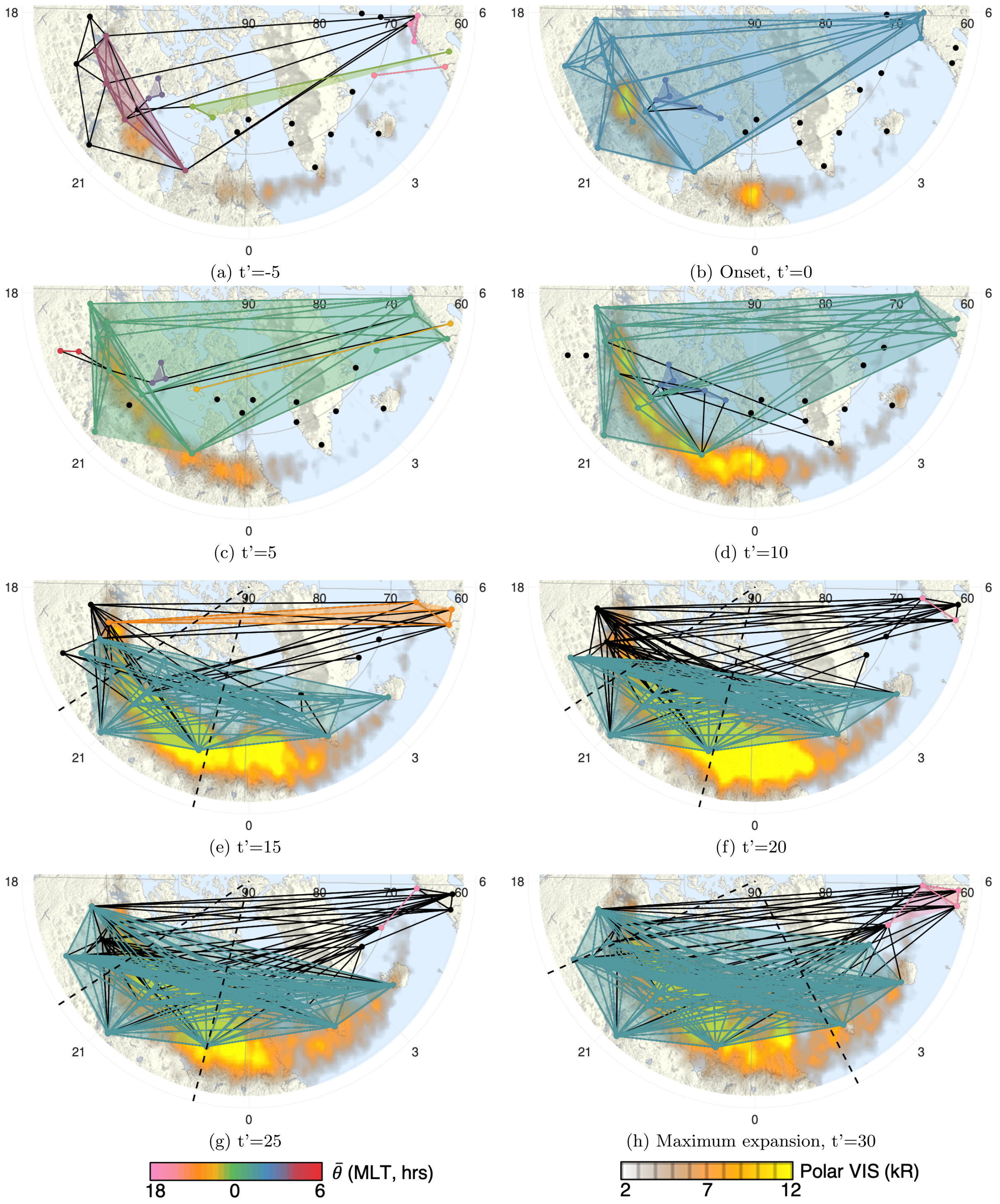

(a) t'=-5
(b) Onset, t'=0
(c) t'=5
(d) t'=10
(e) t'=15
(f) t'=20
(g) t'=25
(h) Maximum expansion, t'=30

Supplementary Figure 13. Community structure snapshots of an example substorm network in normalized time t'. The community structure of a substorm on 20/01/1998, plotted in the same format as in Supplementary Figure 1 (which shows a different event, 16/03/1997). Polar plots are in magnetic coordinates centered at the magnetic pole, where magnetic local time (MLT, hrs) increases clockwise, with midnight located at the bottom (MLT=0 hrs). Maps show the nightside from dusk (MLT=18 hrs) to dawn (MLT=6 hrs) and 60-90° magnetic latitude. The colorbars at the bottom of the figure represent the MLT of the centroid ($\bar{\theta}(t')$) of each community, and polar VIS data from left to right respectively. The network connections are color coded using the left and match those of panels c-d in Supplementary Figure 11. Each subplot (a-h) represents a snapshot of the community structure in intervals of 5 normalized minutes from before onset (panel a, $t' = -5$) to the time of maximum expansion (panel h, $t' = 30$), corresponding to the times in Supplementary Figure 11. The circles represent ground magnetometers with the lines representing network connections. Black magnetometers/connections are not part of a community. The black dashed lines, in panels e-h, are the locations of the auroral bulge found from auroral images. The networks are overplotted on maps provided by superMAG[2] containing polar VIS data[3] in kR, matching the right colorbar. The smaller communities transition into a dominant community, with a single smaller community ($\sim$ 6 MLT) persisting throughout the substorm. Between the communities there are many connections. This plot used the edge betweenness community detection algorithm as in the main text.

Supplementary Note 2

Supplementary Figures 14-22 overlay the (normalized) modularity of multiple substorms in the same format as shown in Figure 3, main text. 14 shows the un-normalized modularity, Q, (in Figure 3, main text, the normalized modularity, Q_N, is overlayed for multiple substorms) using the same detection algorithm, threshold and set of substorms as are used within the main text. 15-19 plot the normalized modularity, Q_N, obtained using the optimal[4], walk trap[5], information mapping[6], leading eigenvector[5] and label propagation[7] methods for community detection, respectively, for the networks calculated from the set of events used in the main paper. 20-22 show the normalized modularity calculated from the networks of a different sets of substorms than those used in the main text, including non-isolated substorms; the edge betweenness algorithm has been used as in the main text. For 23-24 the cross-correlation threshold, at which the raw network was calculated, is varied so that the networks contain more or less connections than those used within the main text; the set of events and community detection method are the same as those used for Figure 3, main text.

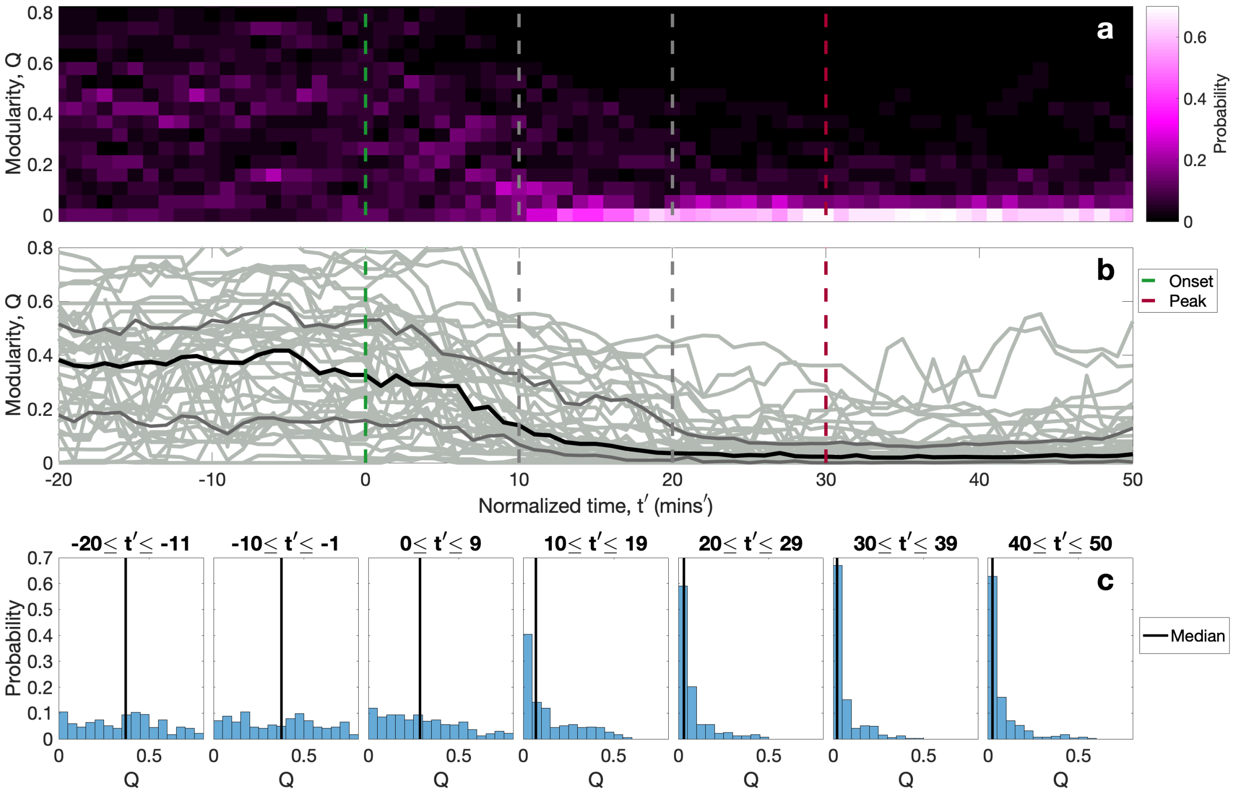

Supplementary Figure 14. Community structure of multiple substorm events. The un-normalized modularity, Q, in the same format as Figure 3, main text (Figure 3 showed the normalized modularity). This plot used the edge betweenness community detection algorithm to calculated the modularity for the 41 isolated substorms, as used in the main text. Panels a-b share normalized time as the abscissa. The panel a ordinate bins Q_N at each normalized time and the color indicates the probability (count of substorms with Q_N/total number of substorms). Panel b plots Q_N of each of the 41 substorms as a function of normalized time, t' , as thin light grey lines. The median is overplotted in black and the 25% and 75% quantiles in darker grey. Panel c plots the normalized histograms of Q_N of the events aggregated over 10 minute intervals as time progresses. The median is overplotted. The substorms have different starting values of modularity which all tend to near zero during and following substorm expansion.

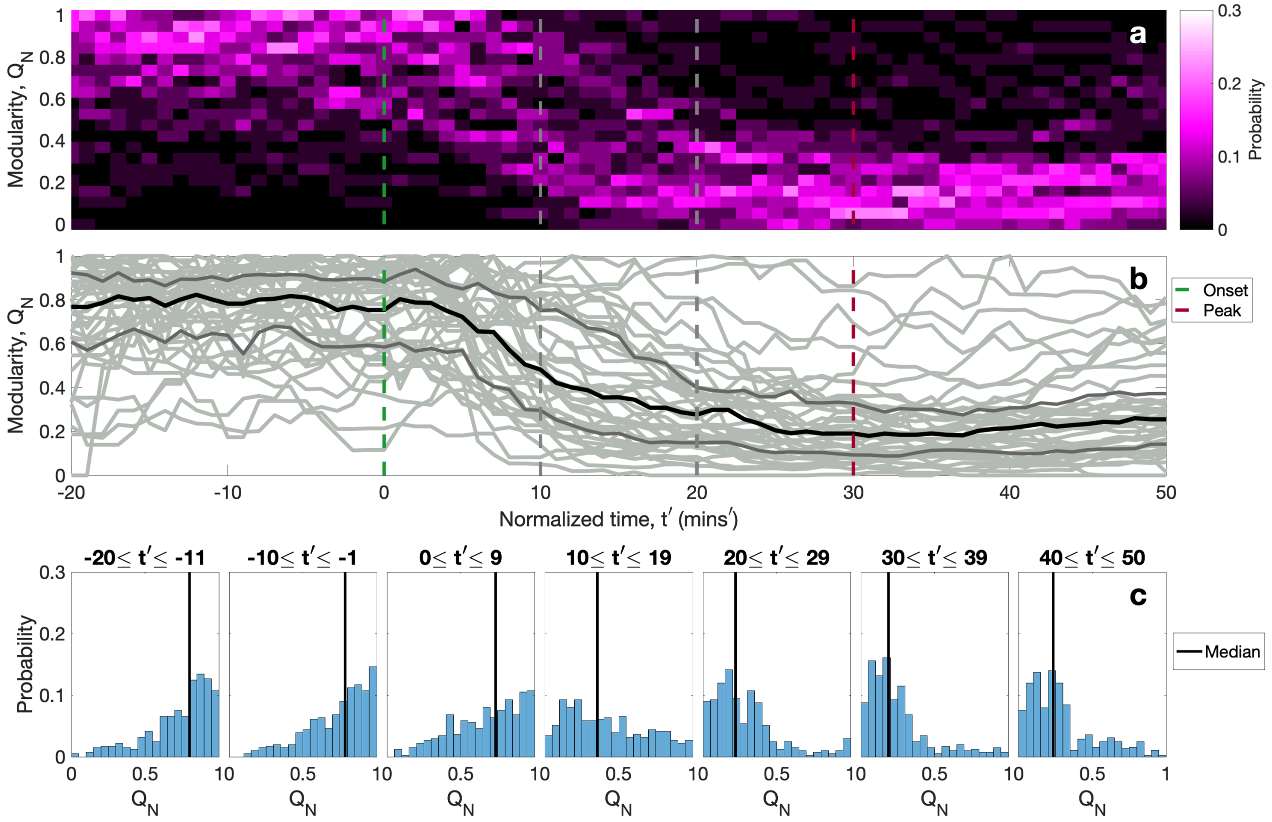

Supplementary Figure 15. Community structure of multiple substorm events. The normalized modularity, Q_N, calculated using the optimal community detection algorithm, in the same format as Figure 3 (in the main text the edge betweenness algorithm is used). This plot was calculated for the 41 isolated substorms, as used in the main text. Panels a-b share normalized time as the abscissa. The panel a ordinate bins Q_N at each normalized time and the color indicates the probability (count of substorms with Q_N/total number of substorms). Panel b plots Q_N of each of the 41 substorms as a function of normalized time, t' , as thin light grey lines. The median is overplotted in black and the 25% and 75% quantiles in darker grey. Panel c plots the normalized histograms of Q_N of the events aggregated over 10 minute intervals as time progresses. The median is overplotted. There is a clear transition from high to low modularity throughout the expansion phase.

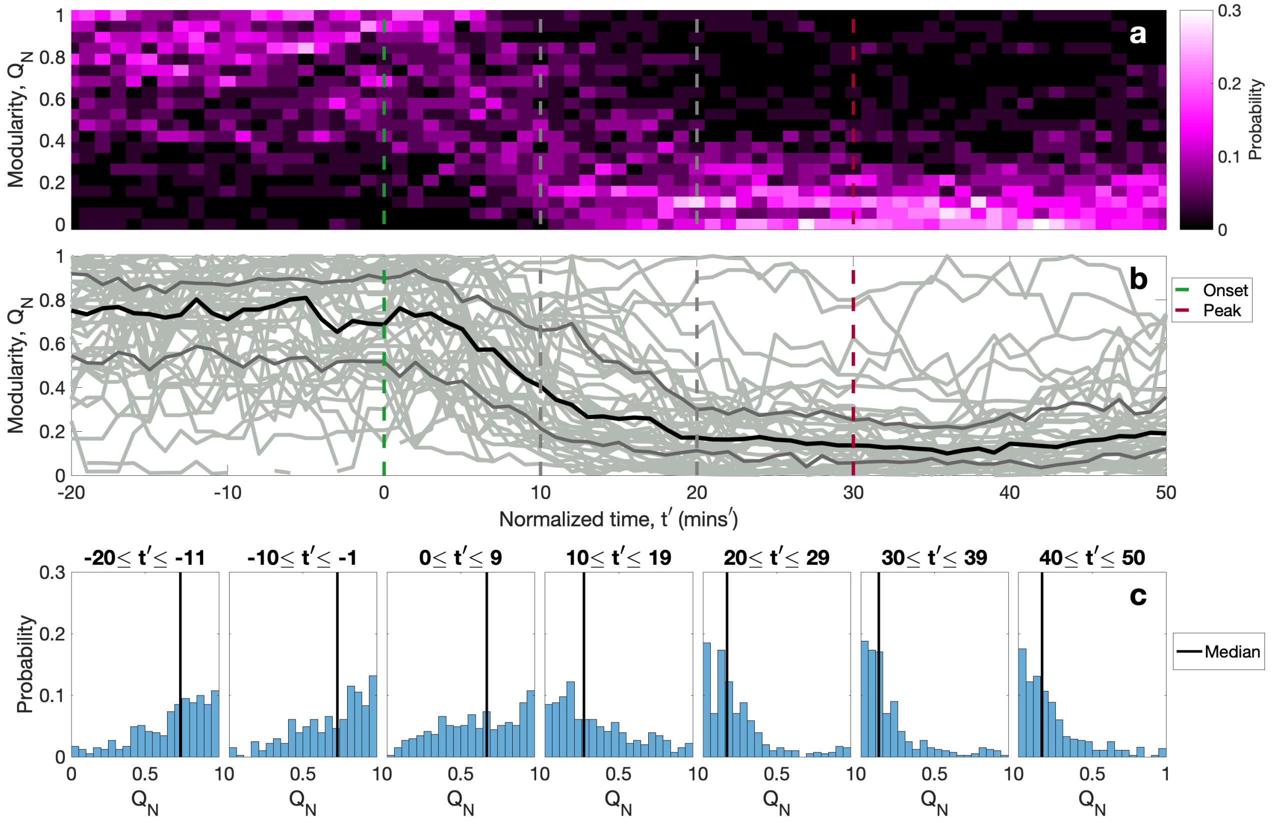

Supplementary Figure 16. Community structure of multiple substorm events. The normalized modularity, Q_N, calculated using the walk trap community detection algorithm, in the same format as Figure 3 (the main text used the edge betweenness algorithm). This plot was calculated for the 41 isolated substorms, as used in the main text. Panels a-b share normalized time as the abscissa. The panel a ordinate bins Q_N at each normalized time and the color indicates the probability (count of substorms with Q_N/total number of substorms). Panel b plots Q_N of each of the 41 substorms as a function of normalized time, t' , as thin light grey lines. The median is overplotted in black and the 25% and 75% quantiles in darker grey. Panel c plots the normalized histograms of Q_N of the events aggregated over 10 minute intervals as time progresses. The median is overplotted. There is a clear transition from high to low modularity throughout the expansion phase.

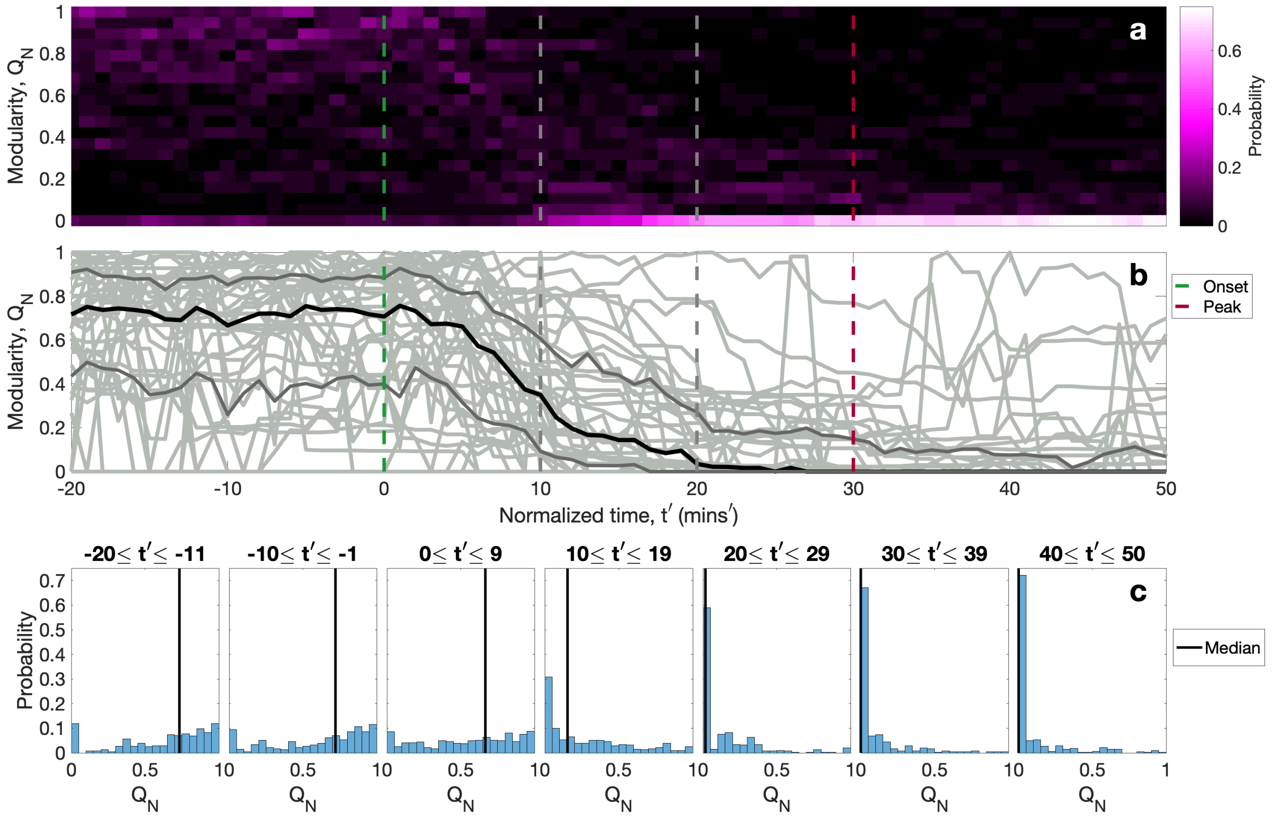

Supplementary Figure 17. Community structure of multiple substorm events. The normalized modularity, Q_N, calculated using the information mapping community detection algorithm, in the same format as Figure 3 (the main text used the edge betweenness algorithm). This plot was calculated for the 41 isolated substorms, as used in the main text. Panels a-b share normalized time as the abscissa. The panel a ordinate bins Q_N at each normalized time and the color indicates the probability (count of substorms with Q_N/total number of substorms). Panel b plots Q_N of each of the 41 substorms as a function of normalized time, t' , as thin light grey lines. The median is overplotted in black and the 25% and 75% quantiles in darker grey. Panel c plots the normalized histograms of Q_N of the events aggregated over 10 minute intervals as time progresses. The median is overplotted. There is a clear transition from high to low modularity throughout the expansion phase.

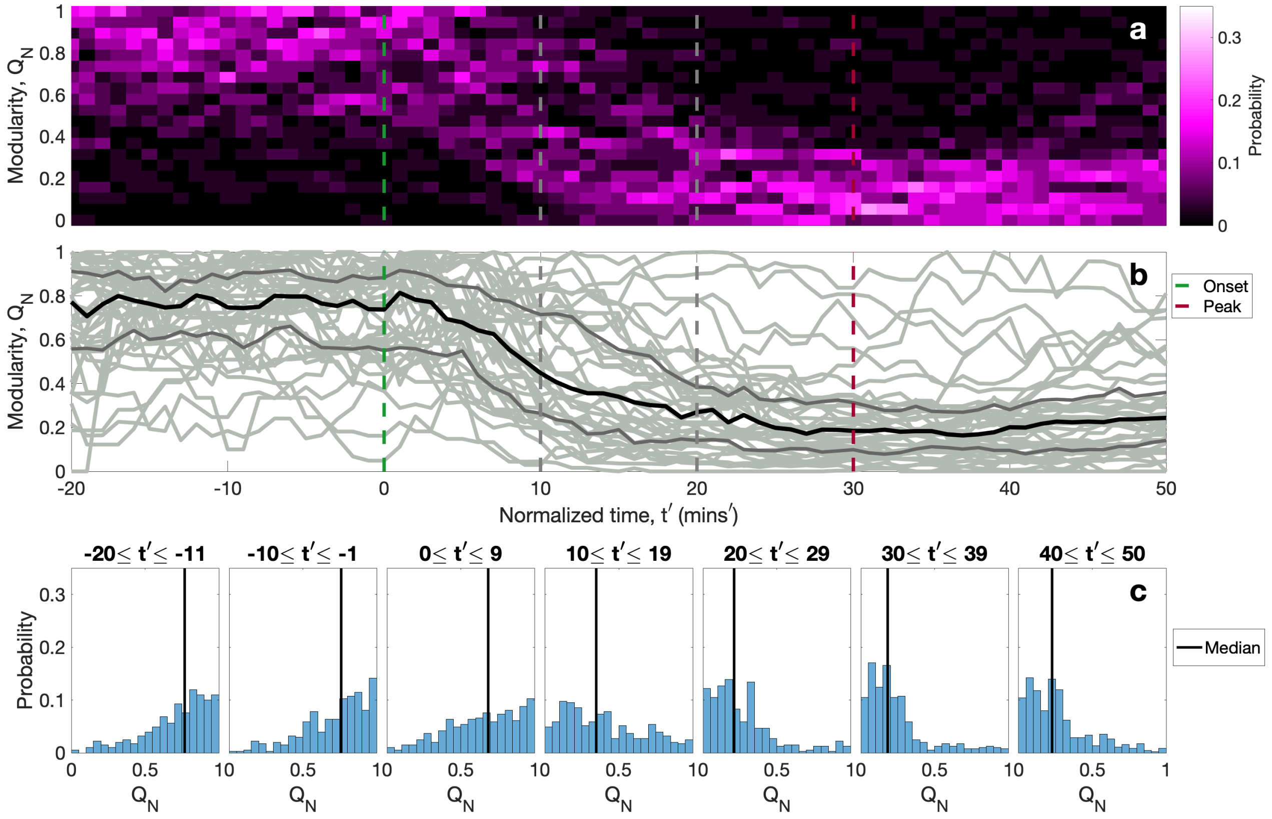

Supplementary Figure 18. Community structure of multiple substorm events. The normalized modularity, Q_N, calculated using the leading eigenvector community detection algorithm, in the same format as Figure 3 (the main text used the edge betweenness algorithm). This plot was calculated for the 41 isolated substorms, analysed in the main text. Panels a-b share normalized time as the abscissa. The panel a ordinate bins Q_N at each normalized time and the color indicates the probability (count of substorms with Q_N/total number of substorms). Panel b plots Q_N of each of the 41 substorms as a function of normalized time, t' , as thin light grey lines. The median is overplotted in black and the 25% and 75% quantiles in darker grey. Panel c plots the normalized histograms of Q_N of the events aggregated over 10 minute intervals as time progresses. The median is overplotted. There is a clear transition from high to low modularity throughout the expansion phase.

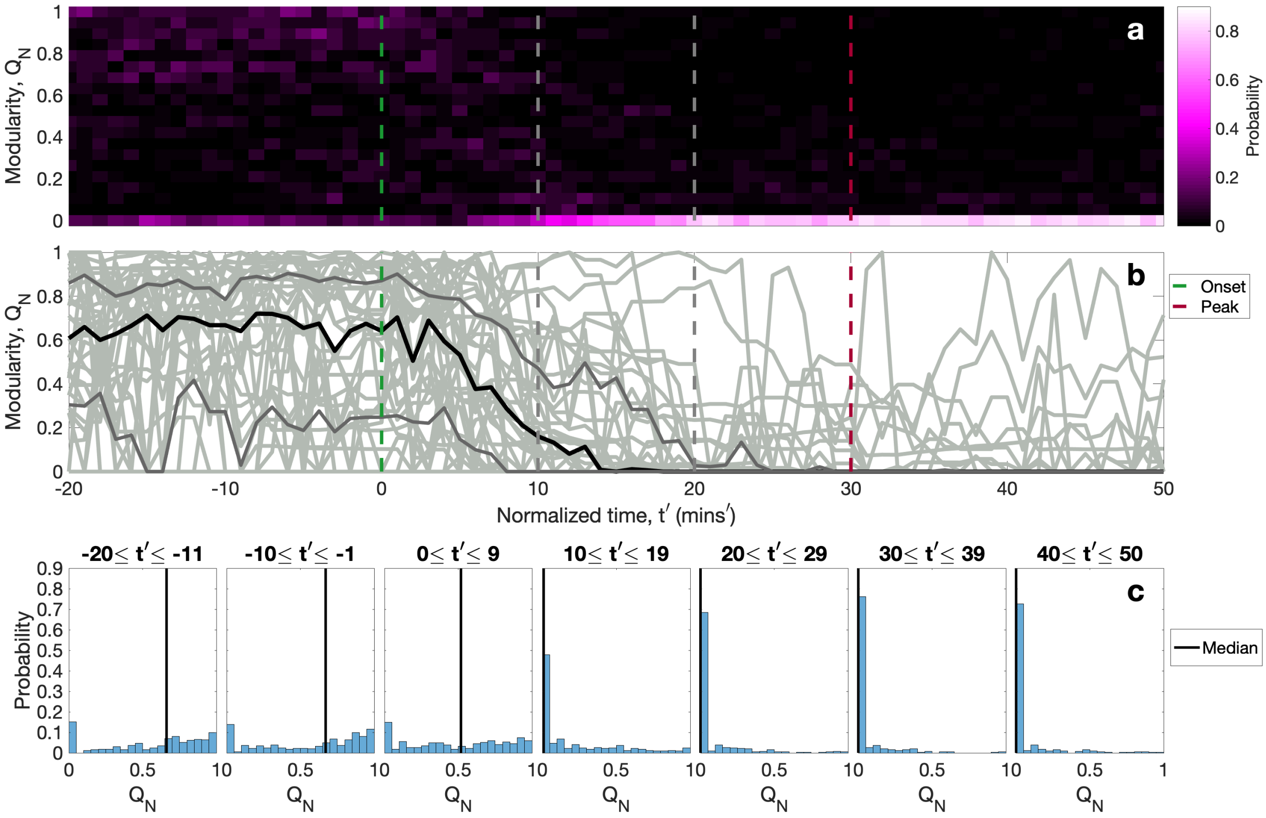

Supplementary Figure 19. Community structure of multiple substorm events. The normalized modularity, Q_N, calculated using the label propagating community detection algorithm, in the same format as Figure 3 (the main text used the edge betweenness algorithm). This plot was calculated for the 41 isolated substorms analysed in the main text. Panels a-b share normalized time as the abscissa. The panel a ordinate bins Q_N at each normalized time and the color indicates the probability (count of substorms with Q_N/total number of substorms). Panel b plots Q_N of each of the 41 substorms as a function of normalized time, t' , as thin light grey lines. The median is overplotted in black and the 25% and 75% quantiles in darker grey. Panel c plots the normalized histograms of Q_N of the events aggregated over 10 minute intervals as time progresses. The median is overplotted. There is a clear transition from high to low modularity throughout the expansion phase.

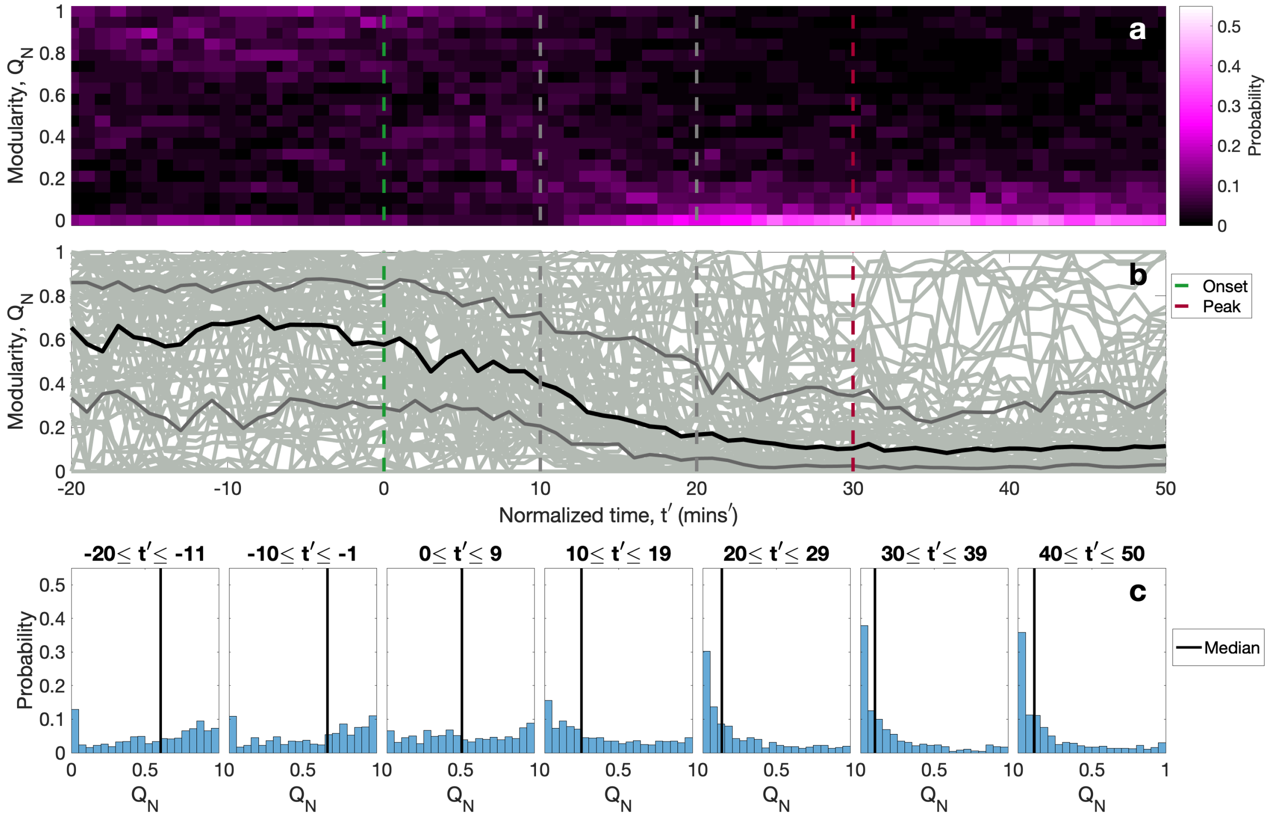

Supplementary Figure 20. Community structure of 75 substorm events. The normalized modularity, Q_N, of 75 substorms with good coverage, in the same format as Figure 3, main text (which presents 41 substorms which were extremely quiet before onset). This plot used the edge betweenness community detection algorithm as in the main text. Panels a-b share normalized time as the abscissa. The panel a ordinate bins Q_N at each normalized time and the color indicates the probability (count of substorms with Q_N/total number of substorms). Panel b plots Q_N of each of the 75 substorms as a function of normalized time, t' , as thin light grey lines. The median is overplotted in black and the 25% and 75% quantiles in darker grey. Panel c plots the normalized histograms of Q_N of the events aggregated over 10 minute intervals as time progresses. The median is overplotted. The transition from high to low modularity throughout the expansion phase is still evident, but there are more outliers, particularly at low modularity before onset.

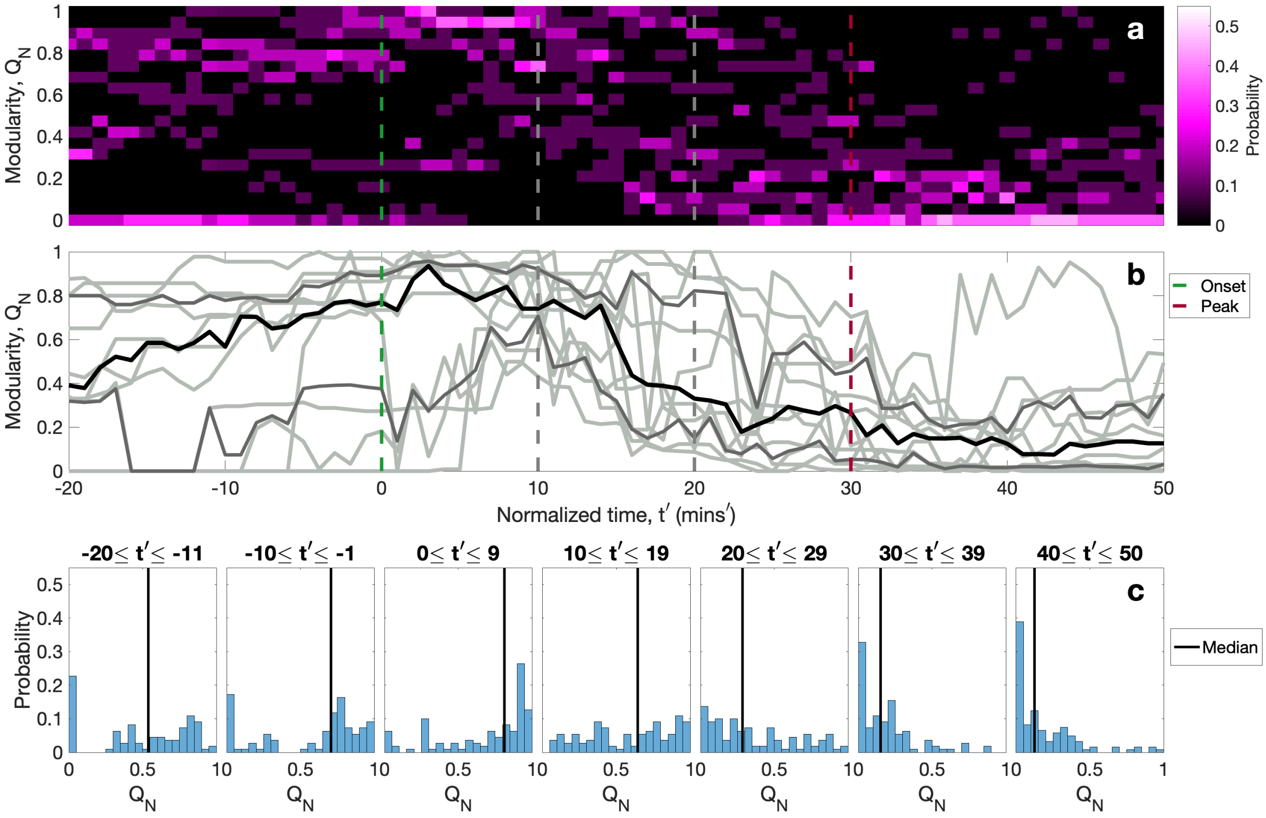

Supplementary Figure 21. Community structure of 11 substorm events. The normalized modularity, Q_N, of 11 substorms with good coverage, that are almost quiet before onset (SML$>$ 25% of that at the time of peak expansion during the 127 minute window before onset but $<$ 50%), in the same format as Figure 3, main text (which contained 41 substorms which were extremely quiet before onset (SML $<$ 25%)). Panels a-b share normalized time as the abscissa. This plot used the edge betweenness community detection algorithm as in main text. The panel a ordinate bins Q_N at each normalized time and the color indicates the probability (count of substorms with Q_N/total number of substorms). Panel b plots Q_N of each of the 11 substorms as a function of normalized time, t' , as thin light grey lines. The median is overplotted in black and the 25% and 75% quantiles in darker grey. Panel c plots the normalized histograms of Q_N of the events aggregated over 10 minute intervals as time progresses. The median is overplotted. The transition from high to low modularity is still evident but with more outliers, particularly before onset.

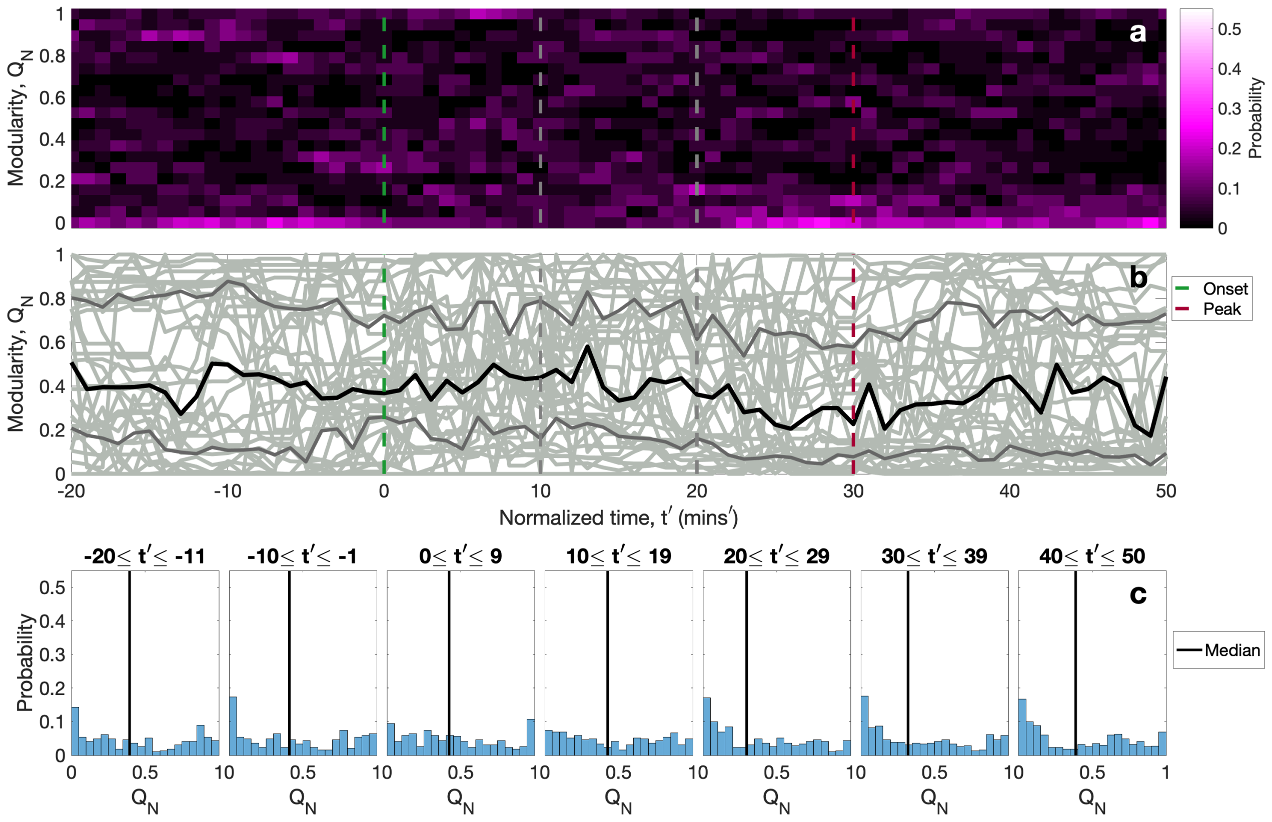

Supplementary Figure 22. Community structure of 23 substorm events. The normalized modularity, Q_N, of 23 substorms with good coverage, that are not quiet before onset, in the format of Figure 3, main text (which presents 41 substorms which were quiet before onset). This plot used the edge betweenness community detection algorithm as in the main text. Panels a-b share normalized time as the abscissa. The panel a ordinate bins Q_N at each normalized time and the color indicates the probability (count of substorms with Q_N/total number of substorms). Panel b plots Q_N of each of the 23 substorms as a function of normalized time, t' , as thin light grey lines. The median is overplotted in black and the 25% and 75% quantiles in darker grey. Panel c plots the normalized histograms of Q_N of the events aggregated over 10 minute intervals as time progresses. The median is overplotted. A transition in modularity is not seen for these non-isolated substorms.

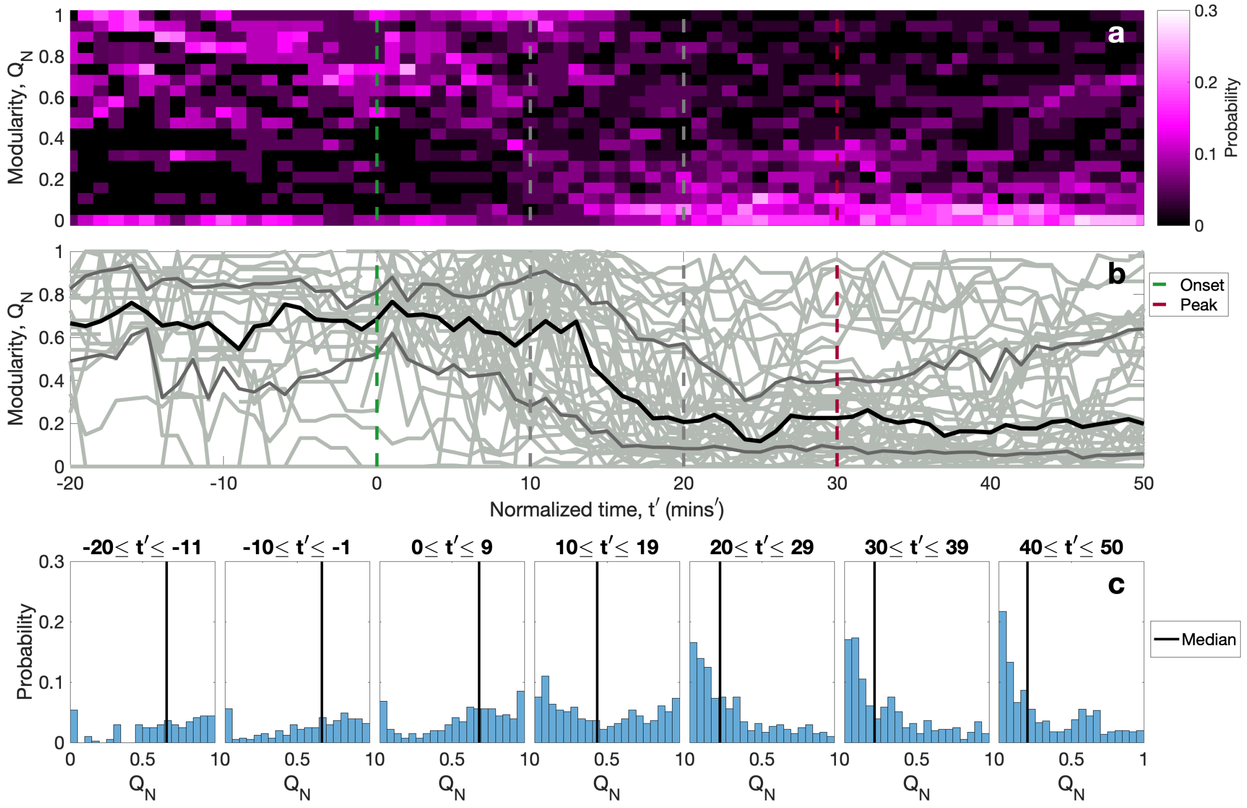

Supplementary Figure 23. Community structure of multiple substorm events with high threshold. The normalized modularity, Q_N, where the network cross-correlation threshold is at a higher value than that used in the main text. Here, station pairs are correlated on average for only 1% of the month surrounding the event, hence fewer connected magnetometers compared to Figure 3, main text (where the threshold was set at 5%). The Figure is in the format of Figure 3, main text and used the edge betweenness community detection algorithm as in the main text. Panels a-b share normalized time as the abscissa. The panel a ordinate bins Q_N at each normalized time and the color indicates the probability (count of substorms with Q_N/total number of substorms). Panel b plots Q_N of each of the 41 substorms as a function of normalized time, t' , as thin light grey lines. The median is overplotted in black and the 25% and 75% quantiles in darker grey. Panel c plots the normalized histograms of Q_N of the events aggregated over 10 minute intervals as time progresses. The median is overplotted. The transition from high to low modularity is still evident, but with more outliers. Before onset, some events exhibit near-zero modularity as the network has few connections.

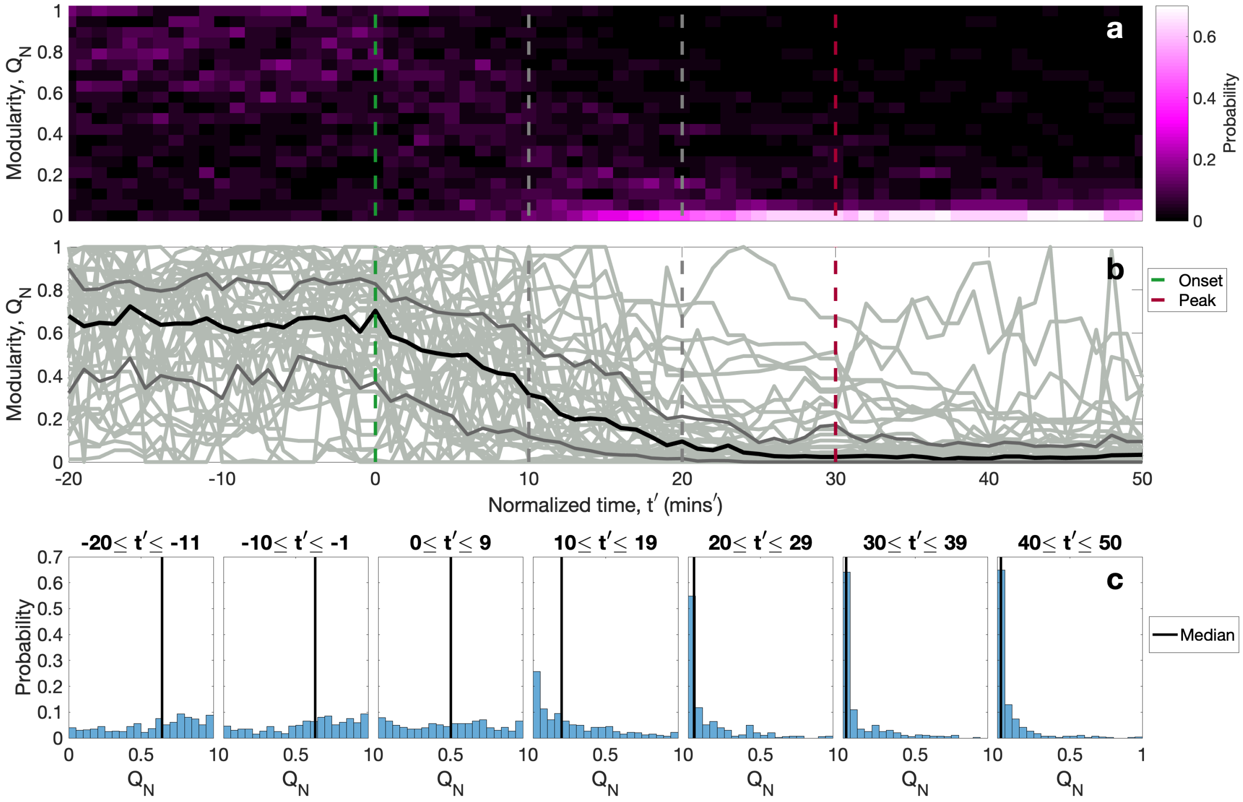

Supplementary Figure 24. Community structure of multiple substorm events with low threshold. The normalized modularity, Q_N, where the network cross-correlation threshold is at a lower value than used in the main text analysis. Here, station pairs are correlated on average for 10% of the month surrounding the event, hence more connected magnetometers compared to Figure 3, main text (where the threshold was set at 5%). The Figure is in the format of Figure 3, main text and used the edge betweenness community detection algorithm as in the main text. Panels a-b share normalized time as the abscissa. The panel a ordinate bins Q_N at each normalized time and the color indicates the probability (count of substorms with Q_N/total number of substorms). Panel b plots Q_N of each of the 41 substorms as a function of normalized time, t' , as thin light grey lines. The median is overplotted in black and the 25% and 75% quantiles in darker grey. Panel c plots the normalized histograms of Q_N of the events aggregated over 10 minute intervals as time progresses. The median is overplotted. The transition from high to low modularity is still evident.

Supplementary Note 3

Supplementary Figure 25 shows how the modularity scales compared to that of a random network with the same degree distribution. The network at each time is randomly rewired while preserving the original graph's degree distribution, using the igraph package[8]. To make a random network the algorithm arbitrarily chooses two edges, (a,b) and (c,d) and substitutes them for (a,d) and (c,b). This is iterated $100 \times N$ times, where N is the number of nodes, to make a random network with the same degree distribution as the original network. We calculate 100 random networks per time point for all 41 substorms, and the modularity is calculated according to the edge betweenness algorithm as per the method in the main text.

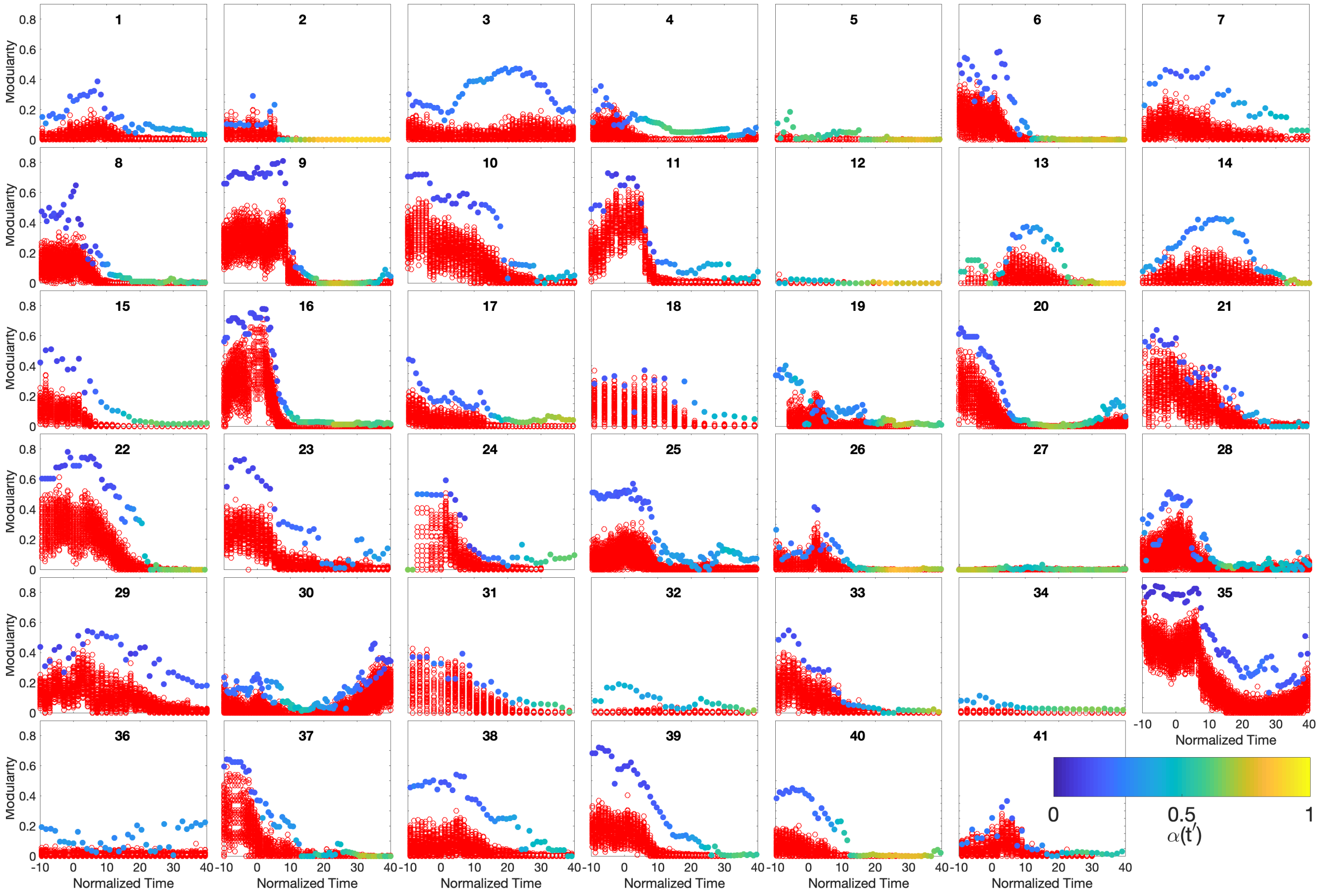

Supplementary Figure 25. Modularity, Q, plotted versus the normalized time, t', for each of the 41 substorms in the main paper. The edge betweenness algorithm has been used for community detection as in main text. The modularity from the networks derived from the observed substorm events is plotted with colour representing the normalized number of connections, or edges, $\alpha(t')$. The red circles plot the modularity obtained from sets of randomly generated networks which have the same degree distribution and number of edges as the observed network at each time. As expected, the random networks explore a range of modularity values (a measure of how separated the communities are) but this range has a clear upper bound. The networks derived from the substorm events explores a broad range of modularity values and this systematically exceeds that of the corresponding random networks that share the same degree distribution and number of edges. Our main result, that the modularity transitions from a high to a low value during substorms, hence does not simply arise as a result of increasing number of network edges with time.

Supplementary Note 4

Supplementary Figure 26 describes the distribution of magnetometers at the time of onset (from polar VIS images) for each of the 41 substorms included in main text. Supplementary Figure 27 is a histogram of the minimum separation distances between magnetometers.

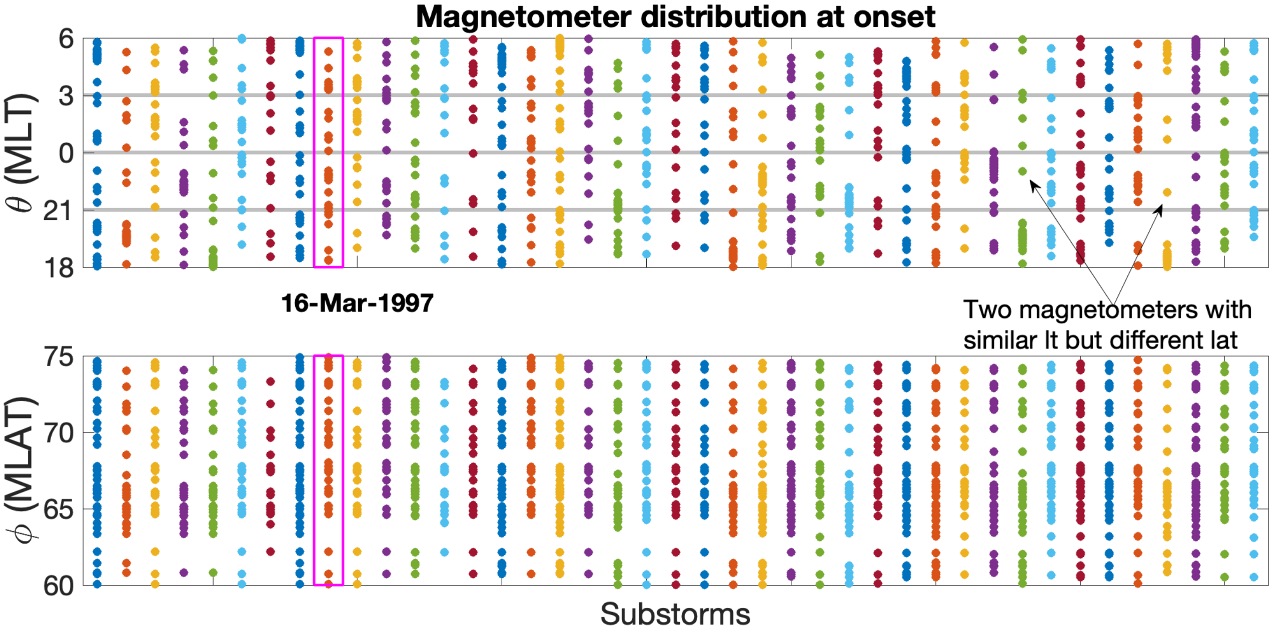

Supplementary Figure 26. Magnetometer distribution at onset. The magnetic local time (top panel) and magnetic latitude (bottom panel) of magnetometers at the time of onset (time determined from polar VIS images[9]) for each of the 41 substorms included in main text. The individual substorm events are arranged along the x axis. Horizontal grey lines separate the nightside into three hour segments in MLT. $\sim$ 90% of 3 hour segments contain more than the minimum of two magnetometers required for analysis.

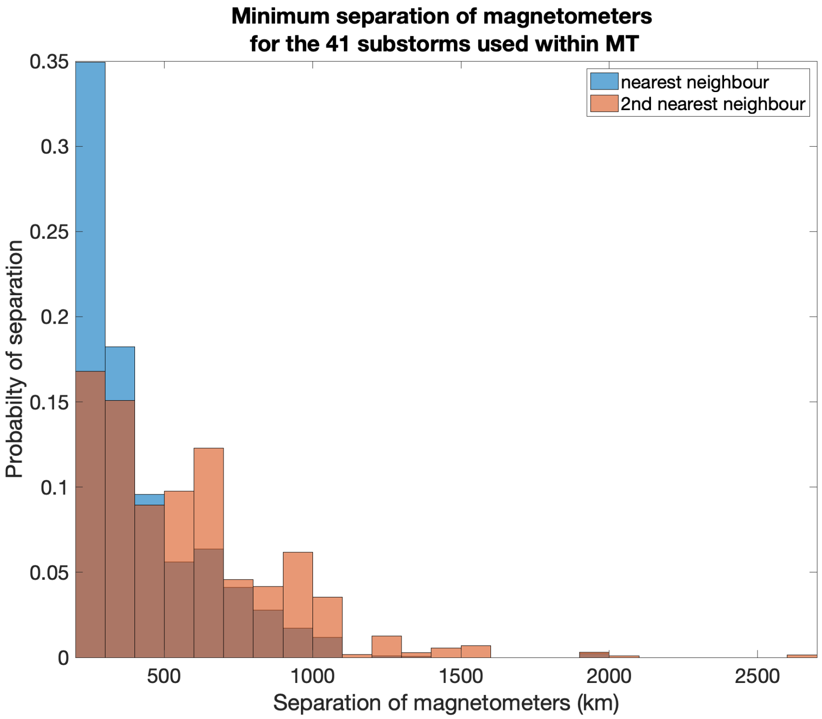

Supplementary Figure 27. A histogram of the geodetic distances between each magnetometer and its two nearest neighbours (for the 41 substorms used in the main text). Separations of > 1000 km are rare.

Supplementary Note 5

Two continuous functions are used to model the magnetic perturbations associated with substorms. The first, $f_1(n)$ represents the DP1 current and the second, $f_2(n)$ represents the DP2 currents. $f(n)$ is given by

$$f_1(t) = -\begin{cases} 0 & t \leq 149, \\ e^{t-179} & 150 \leq t \leq 170, \\ e^{-\frac{t-372}{10}} & 171 \leq 500, \end{cases} \tag{1}$$

$$f_2(t) = -\begin{cases} e^{-(1+(\frac{t-251}{250})^3)^{-1}} & t \leq 250, \\ e^{-(1-(\frac{t-251}{250})^2)^{-1}} & 251 \leq t \leq 500, \end{cases} \tag{2}$$

$$f_3(t) = f_1(t) + f_2(t) \tag{3}$$

The functions are used to approximate the north, B_N, east, B_E, and down, B_Z, components of the vector magnetic field perturbations observed at a magnetometer. Random variables are drawn from the standard normal distribution and multiplied such that noise with the maximum amplitude 30 nT is added to the function; noise is denoted by $N(t)$. The north component is scaled by 400 nT and the east and down components are scaled by 300 nT, e.g. in Supplementary Figure 28, $B_N(t) = N(t) + f_3(t) \times (400 \times 10^{-9})$.

We model the three component vector magnetic field perturbations expected as a response of twelve magnetometers to a substorm using this described method. For each magnetometer pair we then take a window, of length 128, linearly detrend the time series and calculated the canonical cross correlation. This is repeated for each magnetometer pair with a sliding window.

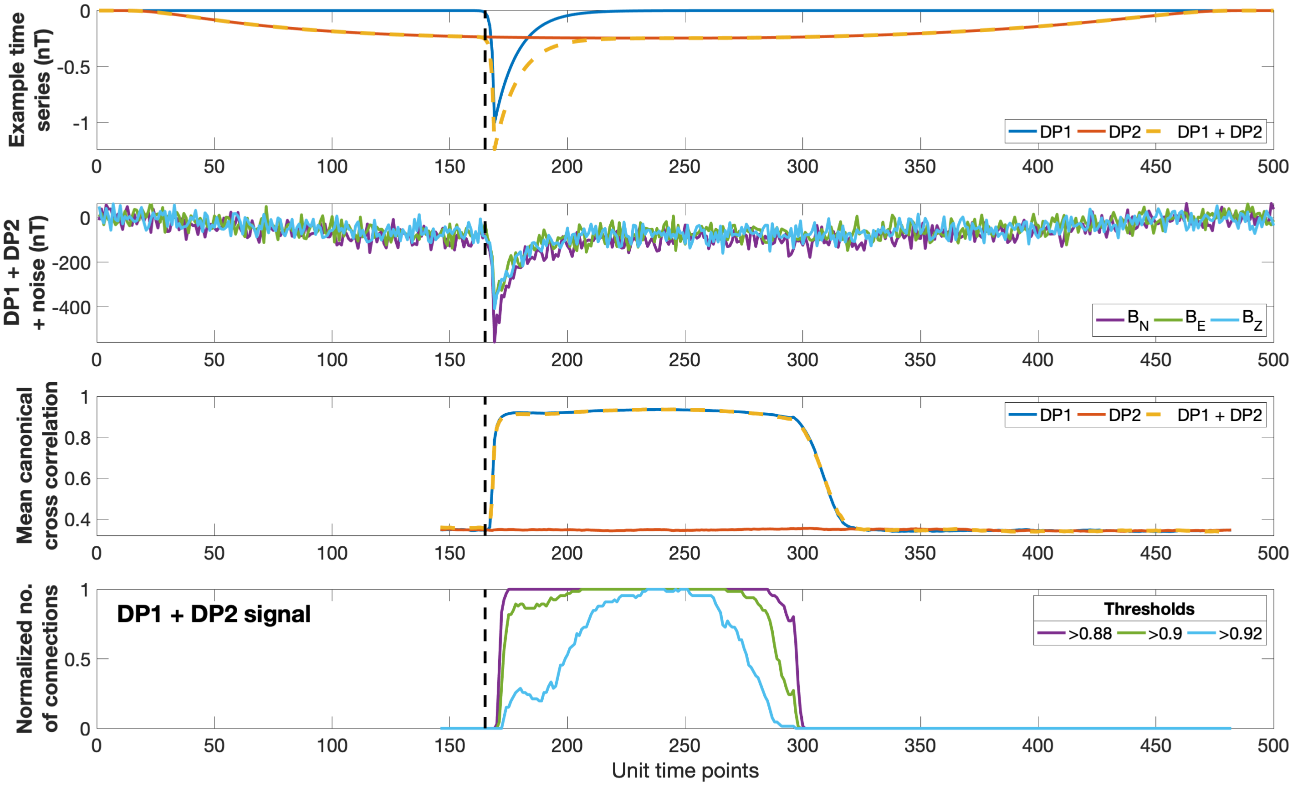

Supplementary Figure 28. Canonical cross correlation for a test set of twelve modelled vector time series. The top panel shows three example time series of the modelled data. DP1 is represented by a sharp exponential increase, and a slower exponential decrease (equation 1). DP2 is represented by a slowly (500 time units) varying curve (equation 2). The second panel shows all three components of the combined DP1 and DP2 currents with noise and scaling. The third panel shows the mean canonical cross correlation across all 12 nodes for the individual currents and the combined current. DP2 shows no increase in correlation throughout whilst the correlation begins to increase within minutes of the sharp decrease for DP1 and the combined current, both of which have a very similar response. The fourth panel shows the normalized number of connections the network would have for different uniform cross correlation thresholds. The network responds as soon as the canonical cross correlation reaches the network threshold.

Supplementary Note 6

Supplementary Figures 29-32 plot the community structure of two flow burst events, the first on the 07/09/2001 is an event studied in[10] and the second on the 05/02/2008 is an event studied in[11].

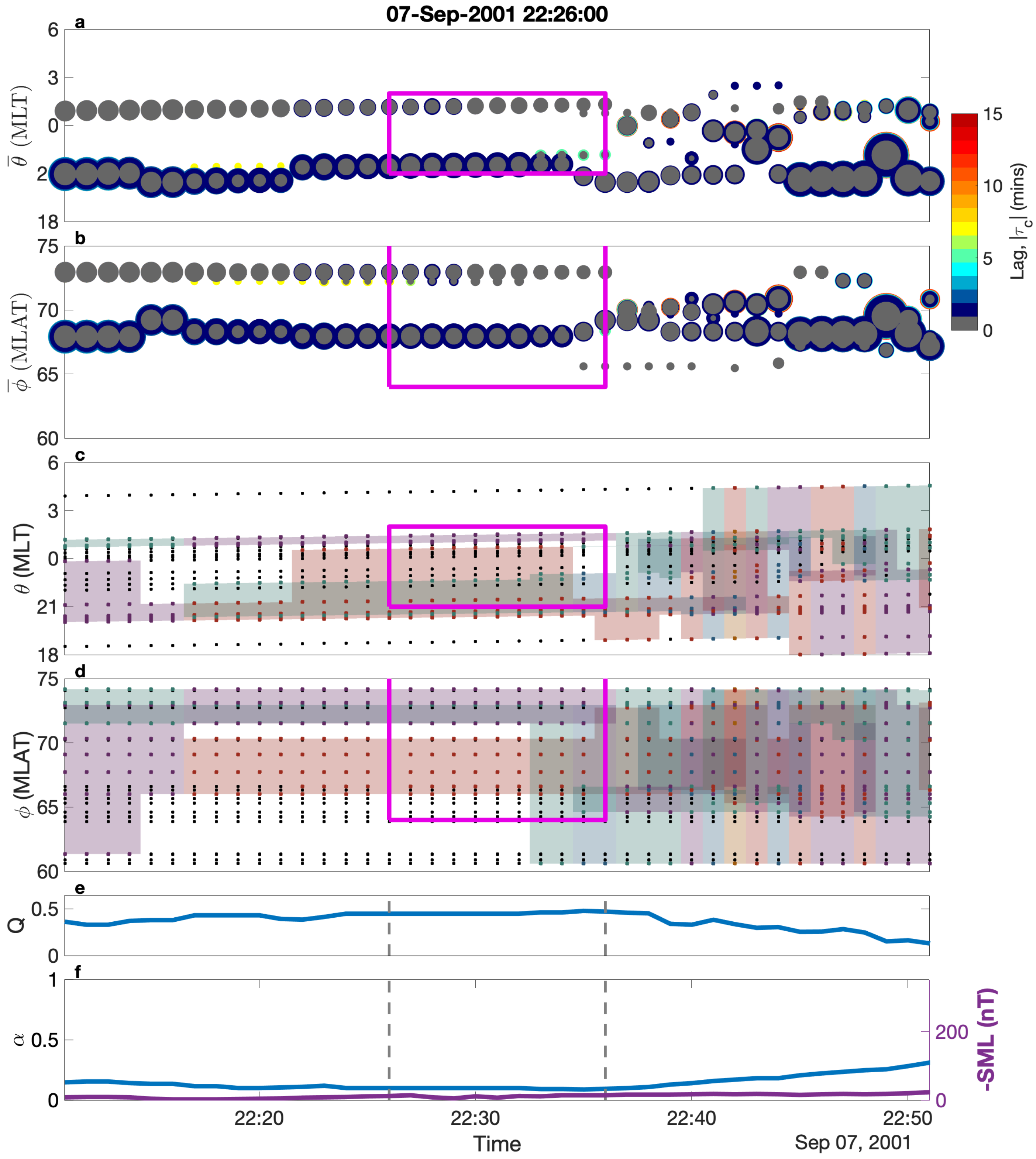

Supplementary Figure 29. The community structure of a flow burst on 07/09/2001 identified in[10]. The abscissa of all panels is universal time. The times and locations highlighted in Figure 2 of[10] are indicated here by pink boxes (panels a-d) and dashed lines (panels e-f). Panels a-b plot the community structure where the size of the circle reflects the normalized number of connections within the community, the ordinate plots the mean MLT/MLAT of the community, $\bar{\theta}_x(t')$ and $\bar{\phi}_x(t')$, and the color indicates the proportion of connections with each lag, $|\tau_c|$. Panels c-d show the spatial extent of each community, where the dots are the specific location of the magnetometers and the shading is the extent. Color identifies individual communities. Black magnetometers are not connected to the network. Panel e plots the modularity, Q. Panel f plots the normalized number of connections, $\alpha(t')$, within the nightside. The right ordinate plots (negative) SML.

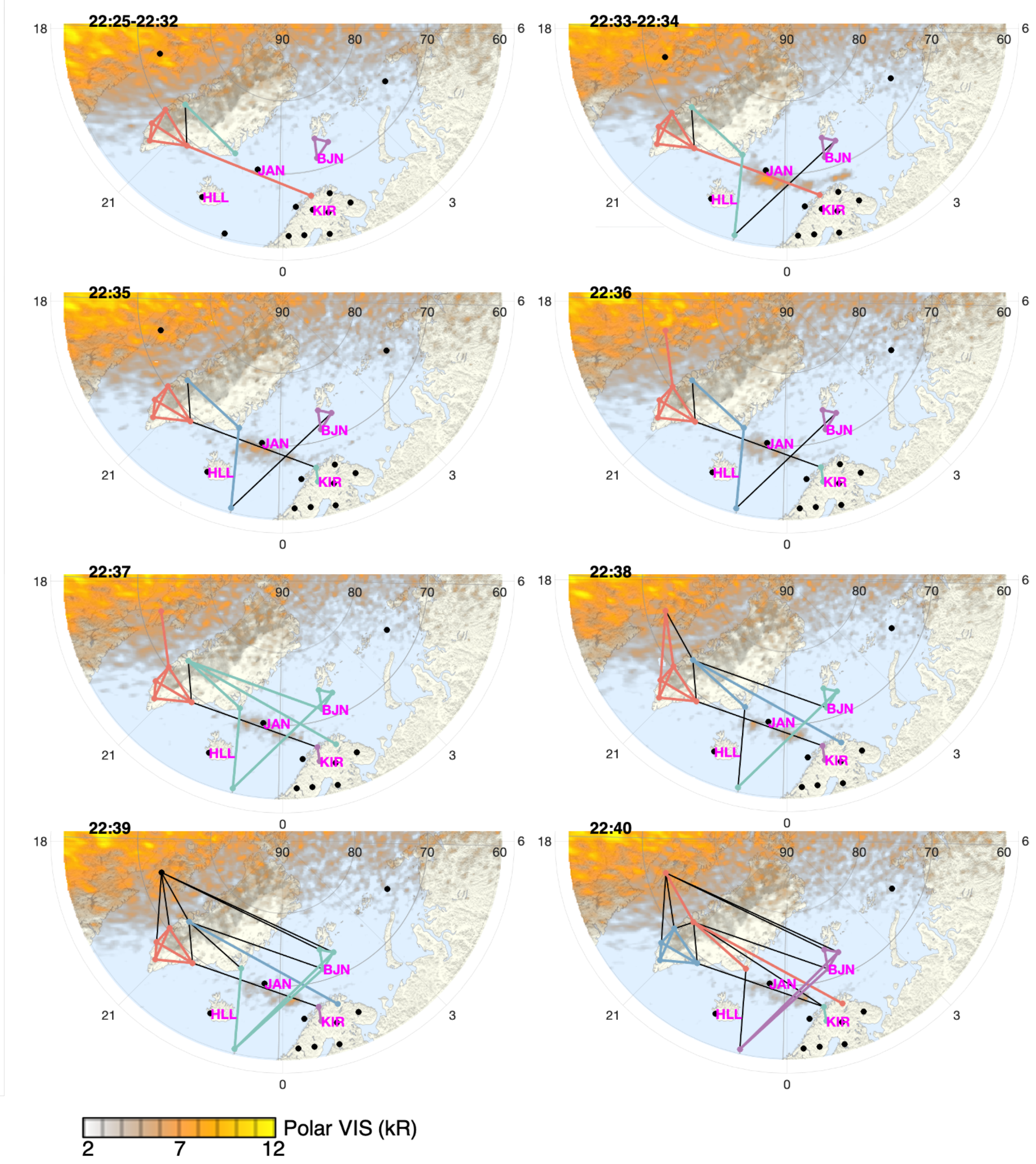

Supplementary Figure 30. The community structure of a flow burst on 07/09/2001 (the event shown in Supplementary Figure 29), is plotted in the same format as Supplementary Figure 1 (which shows a different event, 16/03/1997). Polar plots are in magnetic coordinates centered at the magnetic pole, where magnetic local time (MLT, hrs) increases clockwise, with midnight located at the bottom (MLT=0hrs). Maps show the nightside from dusk (MLT=18 hrs) to dawn (MLT=6 hrs) and 60-90° magnetic latitude. The colorbars at the bottom of the figure represent polar VIS data. The network connections are color coded to each community and match those of panels c-d in Supplementary Figure 29. Each subplot (a-h) represents a snapshot of the community structure in intervals of 5 normalized minutes from before onset (panel a, $t' = -5$) to the time of maximum expansion (panel h, $t' = 30$), corresponding to the times in Supplementary Figure 29. The circles represent ground magnetometers with the lines representing network connections. Black magnetometers/connections are not part of a community. The networks are overplotted on maps provided by superMAG[2] containing polar VIS data[3] in kR, matching the right colorbar. Magnetometers noted in[10] (HLL, JAN, BJN and KIR), Supplementary Figure 2 are labelled here for reference.

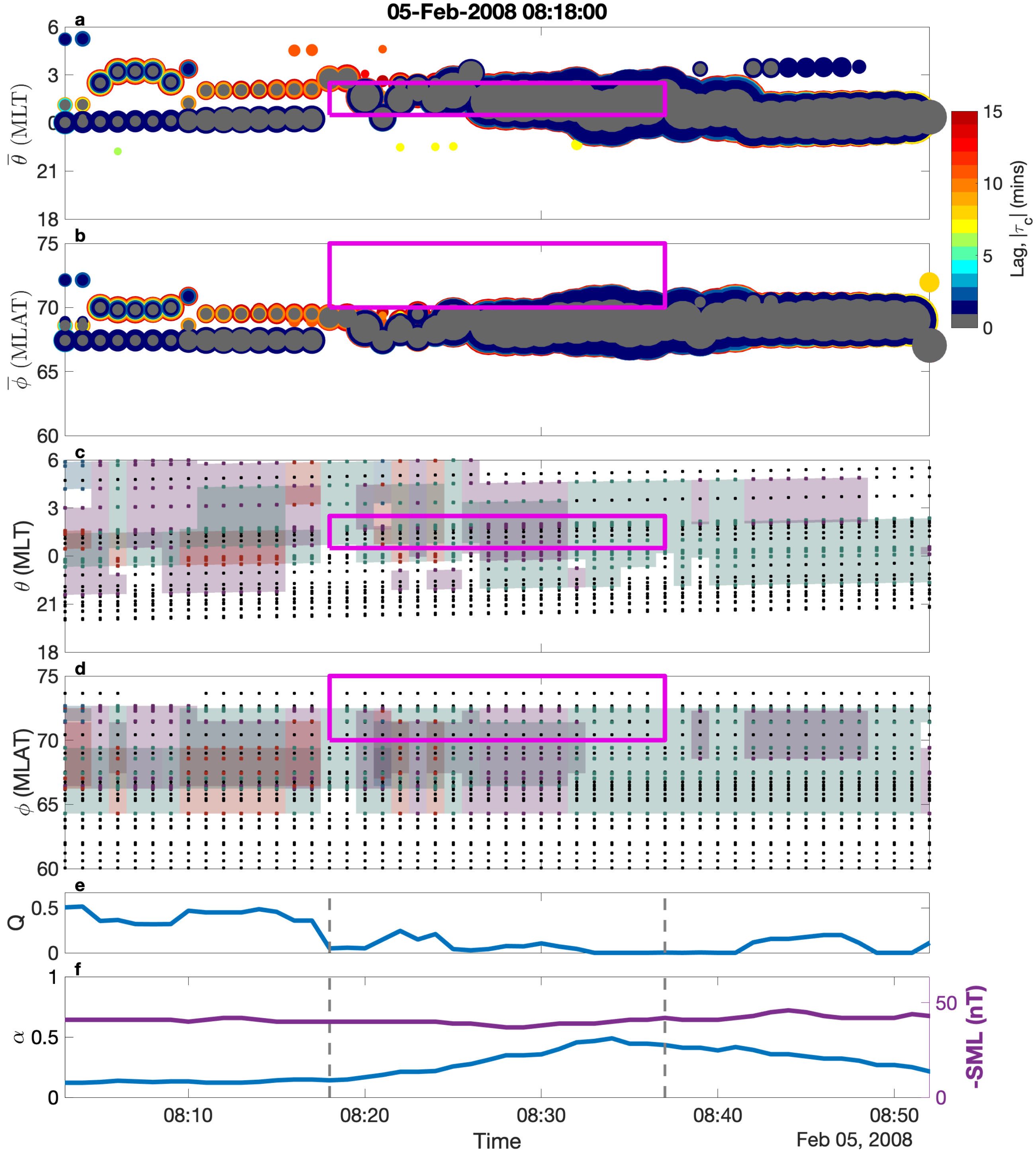

Supplementary Figure 31. The community structure of a flow burst on 05/02/2008, identified in[11] is plotted in the same format as Supplementary Figure 29. The times and locations highlighted in Figure 4 of[11] are indicated here with pink boxes (panels a-d) and dashed lines (panels e-d). Panels a-b plot the community structure where the size of the circle reflects the normalized number of connections within the community, the ordinate plots the mean MLT/MLAT of the community, $\bar{\theta}_x(t')$ and $\bar{\phi}_x(t')$, and the color indicates the proportion of connections with each lag, $|\tau_c|$. Panels c-d show the spatial extent of each community, where the dots are the specific location of the magnetometers and the shading is the extent. Color identifies individual communities. Black magnetometers are not connected to the network. Panel e plots the modularity, Q. Panel f plots the normalized number of connections, $\alpha(t')$, within the nightside. The right ordinate plots (negative) SML.

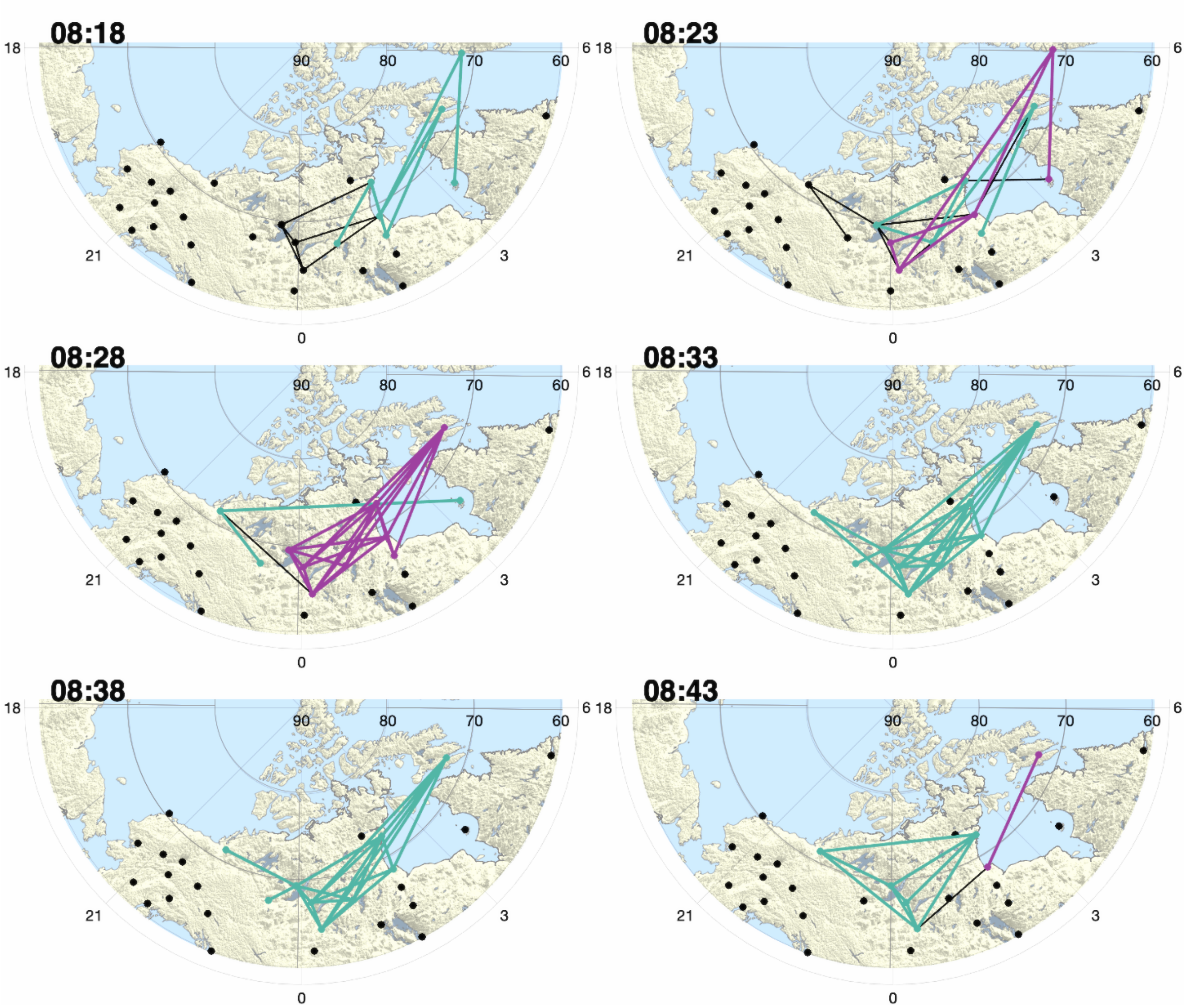

Supplementary Figure 32. The community structure of a flow burst on 05/09/2001 (the event shown in Supplementary Figure 31) is plotted in the same format as Supplementary Figure 1 (which shows a different event, 16/03/1997). Polar plots are in magnetic coordinates centered at the magnetic pole, where magnetic local time (MLT, hrs) increases clockwise, with midnight located at the bottom (MLT=0hrs). Maps show the nightside from dusk (MLT=18 hrs) to dawn (MLT=6 hrs) and 60-90° magnetic latitude. The network connections are color coded to each community and match those of panels c-d in Supplementary Figure 31. Each subplot (a-h) represents a snapshot of the community structure in intervals of 5 normalized minutes from before onset (panel a, $t' = -5$) to the time of maximum expansion (panel h, $t' = 30$), corresponding to the times in Supplementary Figure 31. The circles represent ground magnetometers with the lines representing network connections. Black magnetometers/connections are not part of a community. The networks are overplotted on maps provided by superMAG[2].

74 Supplementary Note 7

A glossary of terms used within the main text.

Network: In their simplest form, networks are a set of nodes joined by edges/connections. Supplementary Figure 33 is an example of an undirected network and a directed network.

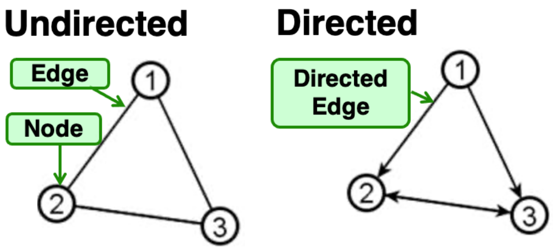

Supplementary Figure 33. A schematic of a undirected (left) and directed (right) network.

Adjacency matrix: If a network contains n nodes, its adjacency matrix, $\mathbf{A}$, is an $n \times n$ matrix where entry a_{ij} will be 1 if vertices i and j are connected (have an edge between them), and 0 otherwise. For an undirected network the matrix is symmetric as there is no associated direction. For a directed network, the matrix is no longer symmetric as a_{ij} represents the directed edge from $i \rightarrow j$ and a_{ji} represents the directed edge from $j \rightarrow i$.

Direction of propagation/expansion: For the directed network in this study, i.e. magnetometers as nodes and directed edges due to time lagged correlation, the direction of propagation/expansion refers to the time lagged correlation between two magnetometers, i.e. if magnetometer i measures a signal and several minutes later magnetometer j measures a highly correlated signal, propagation/expansion is from $i \rightarrow j$. Supplementary Figure 34 shows an example of a lagged connection between two magnetometers.

Canonical correlation: It is often used to study correlations between multivariate datasets[14]. If we have two vector time series, canonical correlation analysis determines the linear combination of the two which are maximally cross-correlated. The cross-correlation between the linear combinations is the (1st) canonical cross-correlation (CCC) component.

Communities: Community structure is a property common to many networks, where the network nodes are divided into groups within which the network connections are dense (vertices are highly interconnected), but between which they are sparser (see Supplementary Figure 35 for an example)[15].

Edge betweenness: The edges between the communities will carry the majority of shortest paths and therefore have the highest edge betweenness. The edge-betweenness algorithm[1] identifies and successively removes the edges which carry the majority of the shortest paths between nodes, i.e. the edges with the highest edge betweenness, leaving behind sub-networks that are the individual communities.

Modularity: If a network is divided into q communities, we define a $q \times q$ symmetric matrix $\mathbf{e}$ whose elements e_{xy} are the fraction of all edges in the network that link nodes in community x to nodes in community y. Then e_{xx} is the fraction of the network contained within community x and e_{xy} is the fraction of the network that connects between communities x and y. The fraction of network edges that connect nodes in community x to the rest of the network is $f_x = \sum_{y=1,q} e_{xy}$. The modularity, Q, is then given by:

$$Q = \sum_{x=1,q} (e_{xx} - f_x^2) \tag{4}$$

Supplementary Figure 35 shows an example of a network with three communities, $q = 3$, and a total of 32 edges. Matrix $\mathbf{e}$ is a 3×3 matrix and the matrix elements are given in Supplementary Figure 35.

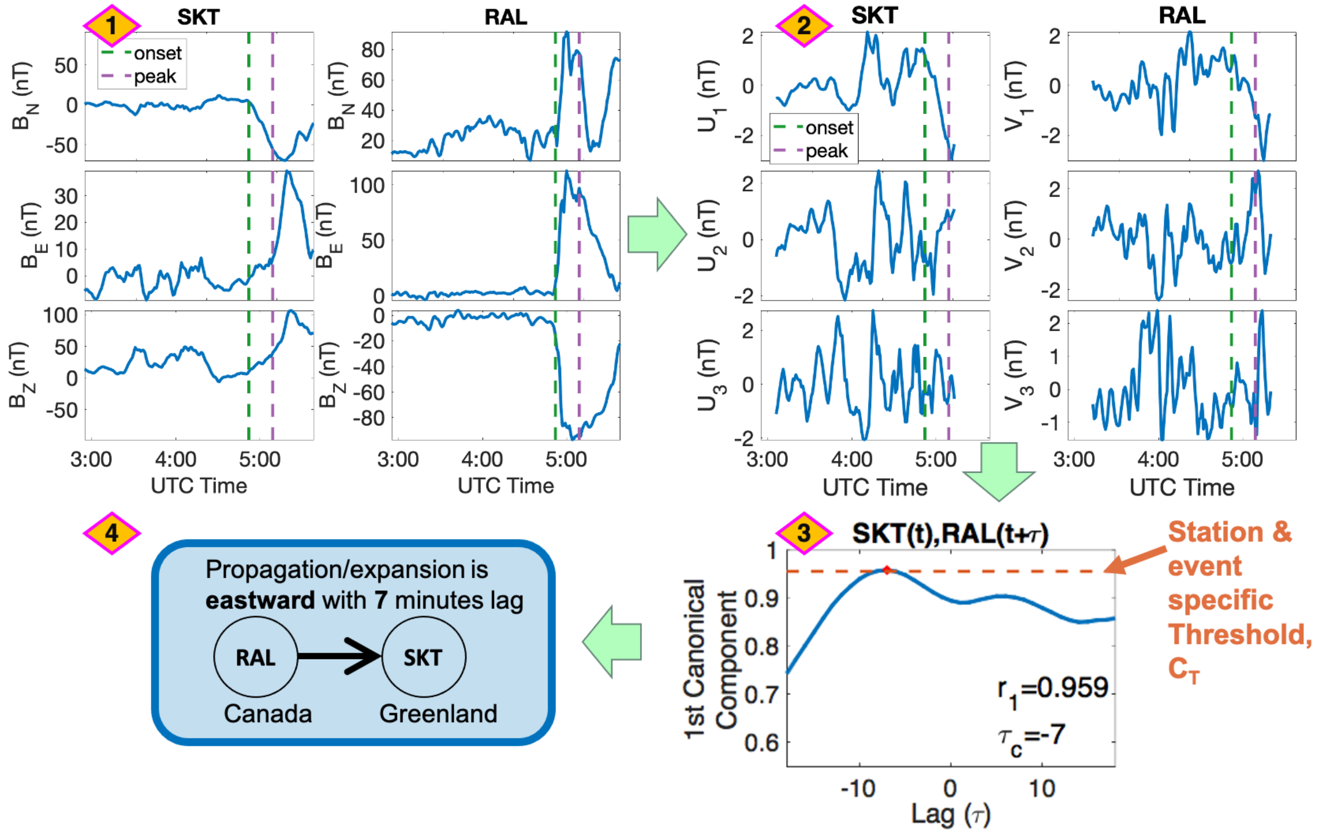

Supplementary Figure 34. A schematic of how a connection/edge between two magnetometers is identified. 1) Take two magnetometer vector magnetic field perturbation time series'. 2) Take a running 128 minute window of each time series, linearly detrended and canonically cross-correlated across positive and negative lags to calculate the linear combination of the two which are maximally correlated (U_1, V_1). 3) If the maximum canonical correlation component (correlation of U_1 and V_1 exceeds the station and event specific threshold[12]), the magnetometers are connected in the network. The cross correlation lag τ_c of that peak value of cross correlation is identified with this network connection. 4) Given the geographical coordinates of the magnetometers we know the direction of propagation/ expansion, in time, of the correlated signal between the two magnetometers[13].

Modularity interpretation: Supplementary Figure 36 contains a schematic of the current system models we would expect from high our low modularity.

Normalized number of connections: The overall network response is parameterized by the normalized number of connections, $\alpha(t)$ (see also[12, 13, 19]). For a directed network α is the simply the number of connections/edges at time t, divided by the the total possible number of connections and is given by,

$$\alpha(t) = \frac{\sum_{i \neq j}^{N(t)} \sum_{j \neq i}^{N(t)} \mathbf{A}_{ij}}{N(t)(N(t)-1)} \quad (5)$$

where $\mathbf{A}$ is the adjacency matrix ($\mathbf{A}_{ij} = 1$ if magnetometers i and j are connected and $\mathbf{A}_{ij} = 0$ otherwise) and $N(t)$ is the number of active magnetometers.

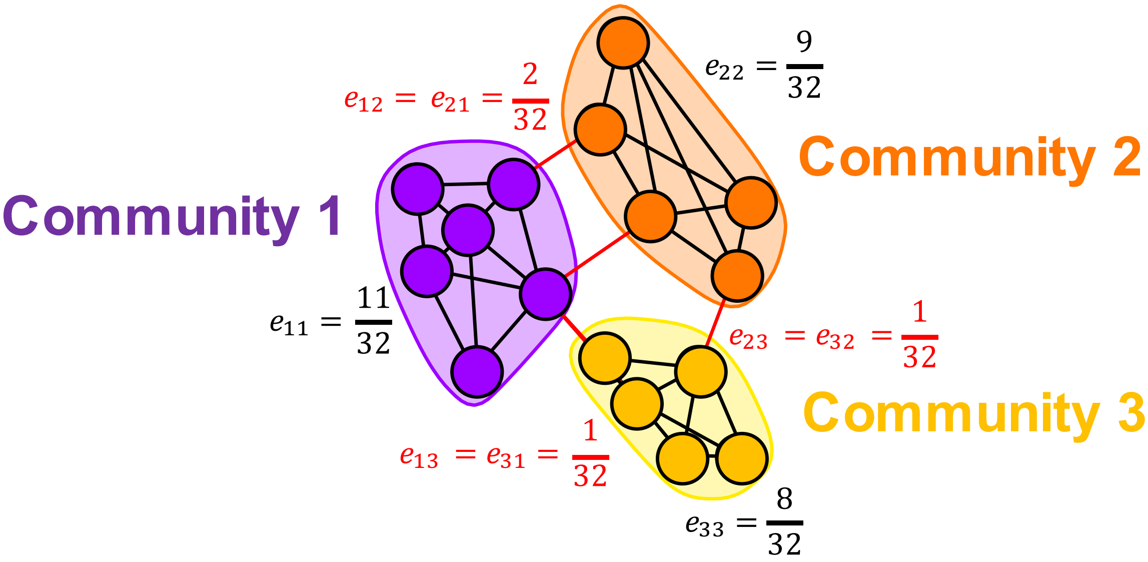

Supplementary Figure 35. A schematic of a network with three communities. Communities are color coded with community 1 in purple, 2 in orange and 3 in yellow. Connections within the communities are black, where as inter-community edges are in red. The numbers are in reference to the matrix elements, e_{xy} from the modularity, equation 4. These numbers are the fraction of edges within (black, e_{xx}) and between (red, e_{xy}) communities.

Time normalization: To compare multiple substorms we first map each event onto a common normalized time-base such that, once normalized, all substorms share a common onset time and take 30 normalized minutes to develop from onset to the maximum expansion. The method for time normalization, t', developed in[9] is as follows:

$$t' = \frac{T_E \times (t - t_{onset})}{t_{peak} - t_{onset}} \tag{6}$$

where $T_E = 30$ minutes, approximately the average length of a substorm expansion phase. The onset time is then at $t' = 0$ and the time of peak expansion $t' = 30$. The critical timings for this normalization, t_{onset} and t_{peak}, can be unambiguously identified in the isolated substorm events used in this paper.

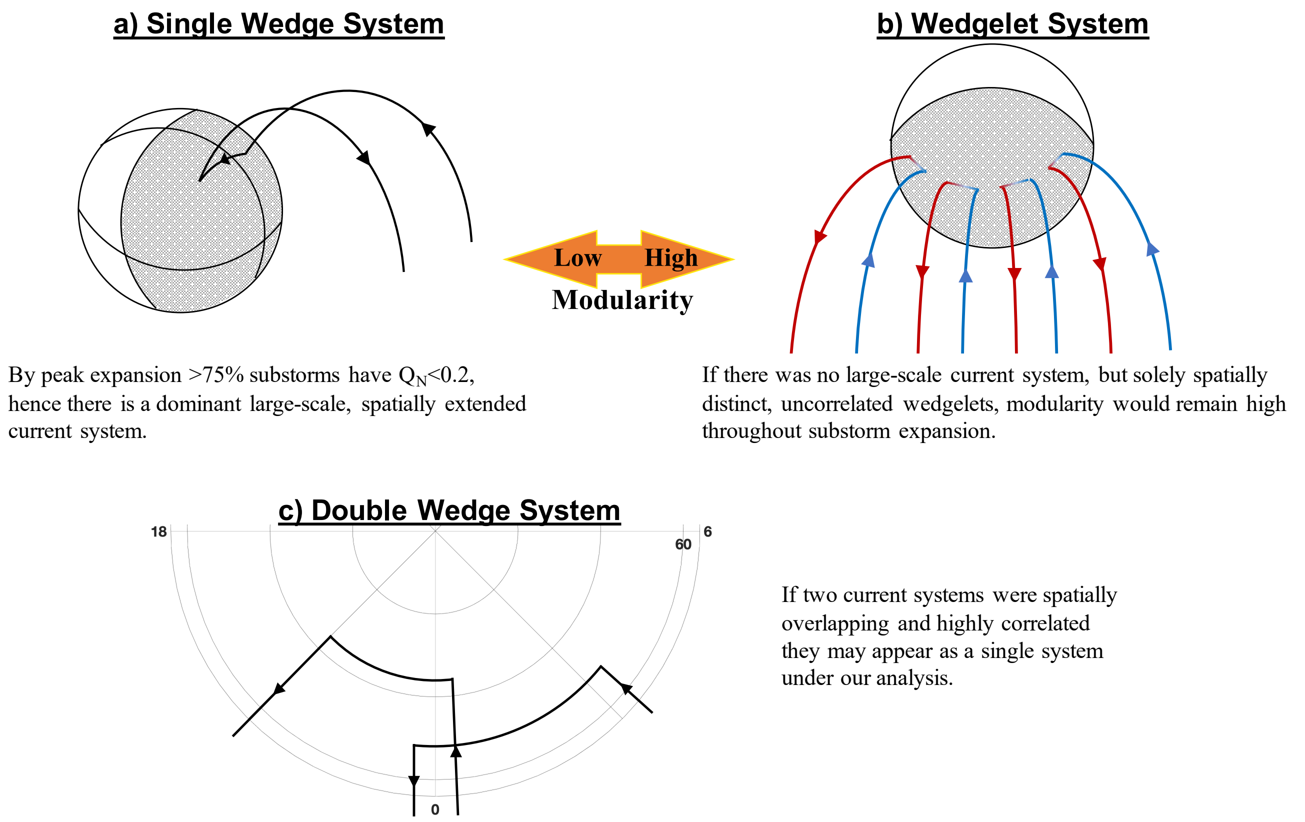

Supplementary Figure 36. A schematic showing our interpretation of high or low modularity. Ionospheric current systems are represented as simple line currents. a) For a single wedge current system, e.g.[16], modularity will be low (near-zero) because all auroral latitude magnetometers are highly correlated due to a large-scale ionospheric current system overhead. b) For a system comprised of individual wedgelets, e.g.[17], the modularity will be high as spatially localized coherent perturbations cause a sub-group/community of magnetometers to be internally correlated, but not cross-correlated with other groups/communities of magnetometers. c) A double or multi-wedge system, e.g.[18], could appear as a single system (low modularity) under our analysis if the current systems were highly correlated and not spatially distinct.

Supplementary Note 8

Supplementary Tables 1-3 give the timings of onset and peak auroral bulge expansion. All substorms listed meet the requirements described in methods, data and event selection section. The 41 events in table 1 have SML index[20] $< 25\%$ of its maximum value (at the peak of the substorm) in the 127 minutes before the start of the substorm. The 11 events in supplementary table 2 have SML$> 25\%$ of that at the time of peak expansion during the 127 minute window before onset but $< 50\%$. The 23 events in supplementary table 3 have SML$> 50\%$ of that at the time of peak expansion during the 127 minute window before onset.

Number	Onset	Peak
1	07-Jan-1997 03:03	07/01/1997 03:32
2	07-Jan-1997 17:29	07/01/1997 17:52
5	27-Jan-1997 10:14	27/01/1997 10:42
6	31-Jan-1997 20:21	31/01/1997 20:59
7	02-Feb-1997 16:06	02/02/1997 16:38
8	24-Feb-1997 08:20	24/02/1997 09:00
9	27-Feb-1997 14:17	27/02/1997 14:36
10	02-Mar-1997 03:29	02/03/1997 04:10
13	16-Mar-1997 05:41	16/03/1997 06:20
16	24-Mar-1997 10:30	24/03/1997 10:57
18	06-Sep-1997 04:57	06/09/1997 05:21
24	06-Nov-1997 04:01	06/11/1997 04:18
26	02-Jan-1998 13:39	02/01/1998 14:08
28	04-Jan-1998 12:37	04/01/1998 13:04
29	06-Jan-1998 02:49	06/01/1998 03:07
31	11-Jan-1998 07:05	11/01/1998 08:05
33	20-Jan-1998 03:42	20/01/1998 04:10
38	17-Mar-1998 10:57	17/03/1998 11:07
40	02-Nov-1998 18:48	02/11/1998 19:40
46	03-Dec-1998 07:11	03/12/1998 07:56
48	05-Dec-1998 11:42	05/12/1998 12:08
50	07-Dec-1998 11:37	07/12/1998 12:12
51	07-Dec-1998 16:12	07/12/1998 16:35
52	12-Dec-1998 20:31	12/12/1998 20:52
54	20-Dec-1998 04:06	20/12/1998 05:05
62	10-Jan-1999 08:46	10/01/1999 09:20
78	29-Oct-1999 19:06	29/10/1999 19:48
80	06-Nov-1999 11:20	06/11/1999 12:17
81	07-Nov-1999 01:45	07/11/1999 02:06
82	25-Dec-1999 05:17	25/12/1999 06:20
87	08-Jan-2000 10:31	08/01/2000 10:45
88	09-Jan-2000 21:32	09/01/2000 21:49
91	25-Jan-2000 17:39	25/01/2000 17:59
99	20-Feb-2000 06:46	20/02/2000 07:02
101	26-Feb-2000 06:01	26/02/2000 06:45
103	06-Mar-2000 04:45	06/03/2000 05:07
104	17-Mar-2000 09:36	17/03/2000 10:12
106	18-Dec-2000 16:06	18/12/2000 16:32
108	29-Dec-2000 03:39	29/12/2000 04:10
113	06-Sep-2001 06:27	06/09/2001 06:58
114	21-Oct-2001 07:15	21/10/2001 07:35

Supplementary Table 1. 41 events used in main text.

Number	Onset	Peak
4	22-Jan-1997 05:05	22/01/1997 05:35
11	03-Mar-1997 16:21	03/03/1997 16:38
35	06-Feb-1998 16:41	06/02/1998 17:44
43	26-Nov-1998 08:10	26/11/1998 08:54
65	18-Jan-1999 13:01	18/01/1999 13:21
74	26-Mar-1999 08:56	26/03/1999 09:39
77	21-Oct-1999 15:55	21/10/1999 16:19
79	02-Nov-1999 00:13	02/11/1999 00:34
83	29-Dec-1999 15:41	29/12/1999 17:07
86	07-Jan-2000 16:05	07/01/2000 16:30
115	31-Oct-2001 07:30	31/10/2001 07:45

Supplementary Table 2. 11 events used in Supplementary Figure 21, Note 2.

Number	Onset	Peak
20	30-Sep-1997 08:15	30/09/1997 08:26
25	16-Dec-1997 21:29	16/12/1997 21:56
30	10-Jan-1998 15:57	10/01/1998 16:56
34	29-Jan-1998 16:02	29/01/1998 16:14
36	21-Feb-1998 19:53	21/02/1998 20:32
39	15-Sep-1998 06:40	15/09/1998 07:00
41	16-Nov-1998 02:28	16/11/1998 03:04
42	16-Nov-1998 21:35	16/11/1998 22:30
44	26-Nov-1998 16:16	26/11/1998 16:54
49	05-Dec-1998 13:51	05/12/1998 14:32
53	16-Dec-1998 10:32	16/12/1998 11:37
57	01-Jan-1999 04:52	01/01/1999 05:09
61	08-Jan-1999 20:03	08/01/1999 20:50
70	03-Feb-1999 21:55	03/02/1999 22:21
72	15-Feb-1999 01:51	15/02/1999 02:07
84	03-Jan-2000 03:08	03/01/2000 03:38
85	03-Jan-2000 19:34	03/01/2000 20:00
95	09-Feb-2000 16:20	09/02/2000 17:00
97	16-Feb-2000 12:05	16/02/2000 12:41
98	19-Feb-2000 10:13	19/02/2000 10:23
100	25-Feb-2000 11:53	25/02/2000 12:07
102	29-Feb-2000 07:27	29/02/2000 07:47
109	03-Jan-2001 12:44	03/01/2001 12:55

Supplementary Table 3. 23 events used in Supplementary Figure 22, Note 2.

References

1. Newman, M. E. & Girvan, M. Finding and evaluating community structure in networks. *Phys. review E* **69**, 026113 (2004).
2. Gjerloev, J. The supermag data processing technique. *J. Geophys. Res. Space Phys.* **117** (2012).
3. Frank, L. *et al.* The visible imaging system (vis) for the polar spacecraft. *Space Sci. Rev.* **71**, 297–328 (1995).
4. Brandes, U. *et al.* On modularity clustering. *IEEE transactions on knowledge data engineering* **20**, 172–188 (2007).
5. Pons, P. & Latapy, M. Computing communities in large networks using random walks. In *International symposium on computer and information sciences*, 284–293 (Springer, 2005).
6. Rosvall, M. & Bergstrom, C. T. Maps of information flow reveal community structure in complex networks. *arXiv preprint physics.soc-ph/0707.0609* (2007).
7. Raghavan, U. N., Albert, R. & Kumara, S. Near linear time algorithm to detect community structures in large-scale networks. *Phys. review E* **76**, 036106 (2007).
8. Csardi, G. & Nepusz, T. The igraph software package for complex network research. *InterJournal* **Complex Systems**, 1695 (2006).
9. Gjerloev, J., Hoffman, R., Sigwarth, J. & Frank, L. Statistical description of the bulge-type auroral substorm in the far ultraviolet. *J. Geophys. Res. Space Phys.* **112** (2007).
10. Grocott, A. *et al.* Multi-instrument observations of the ionospheric counterpart of a bursty bulk flow in the near-earth plasma sheet. (2004).
11. Gallardo-Lacourt, B. *et al.* Coordinated superdarn themis asi observations of mesoscale flow bursts associated with auroral streamers. *J. Geophys. Res. Space Phys.* **119**, 142–150 (2014).
12. Dods, J., Chapman, S. & Gjerloev, J. Network analysis of geomagnetic substorms using the supermag database of ground-based magnetometer stations. *J. Geophys. Res. Space Phys.* **120**, 7774–7784 (2015).
13. Orr, L., Chapman, S. & Gjerloev, J. Directed network of substorms using supermag ground-based magnetometer data. *Geophys. Res. Lett.* **46**, 6268–6278 (2019).
14. Reinsel, G. C. *Review of Canonical Correlations in multivariate analysis*, 68–70 (Springer Science & Business Media, 2003).
15. Newman, M. E. The structure and function of complex networks. *SIAM review* **45**, 167–256 (2003).
16. McPherron, R. L., Russell, C. T. & Aubry, M. P. Satellite studies of magnetospheric substorms on august 15, 1968: 9. phenomenological model for substorms. *J. Geophys. Res.* **78**, 3131–3149 (1973).
17. Liu, J. *et al.* The current system of dipolarizing flux bundles and their role as wedgelets in the substorm current wedge. *Electr. Curr. Geosp. Beyond* 323–337 (2018).
18. Gjerloev, J. & Hoffman, R. The large-scale current system during auroral substorms. *J. Geophys. Res. Space Phys.* **119**, 4591–4606 (2014).
19. Dods, J., Chapman, S. & Gjerloev, J. Characterizing the ionospheric current pattern response to southward and northward imf turnings with dynamical supermag correlation networks. *J. Geophys. Res. Space Phys.* **122**, 1883–1902 (2017).
20. Newell, P. & Gjerloev, J. Evaluation of supermag auroral electrojet indices as indicators of substorms and auroral power. *J. Geophys. Res. Space Phys.* **116** (2011).
